# Mechanical ventilation amplifies intratracheal lipopolysaccharide-induced plasma and brainstem inflammation in preterm foetal sheep

**DOI:** 10.1093/braincomms/fcaf441

**Published:** 2025-11-14

**Authors:** Kayla Vidinopoulos, Zahrah Azman, Ainsley Somers, Sharmony B Kelly, Zoe Johnson, Valerie A Zahra, Alison Thiel, Hui Lu, Eric Herlenius, Nhi T Tran, Stuart B Hooper, Beth J Allison, Robert Galinsky, Graeme R Polglase

**Affiliations:** The Ritchie Centre, Hudson Institute of Medical Research, Melbourne, Victoria, 3165, Australia; Department of Obstetrics and Gynaecology, Monash University, Melbourne, Victoria, 3168, Australia; The Ritchie Centre, Hudson Institute of Medical Research, Melbourne, Victoria, 3165, Australia; Department of Obstetrics and Gynaecology, Monash University, Melbourne, Victoria, 3168, Australia; The Ritchie Centre, Hudson Institute of Medical Research, Melbourne, Victoria, 3165, Australia; Department of Obstetrics and Gynaecology, Monash University, Melbourne, Victoria, 3168, Australia; The Ritchie Centre, Hudson Institute of Medical Research, Melbourne, Victoria, 3165, Australia; The Ritchie Centre, Hudson Institute of Medical Research, Melbourne, Victoria, 3165, Australia; Department of Paediatrics, Monash University, Melbourne, Victoria, 3168, Australia; The Ritchie Centre, Hudson Institute of Medical Research, Melbourne, Victoria, 3165, Australia; The Ritchie Centre, Hudson Institute of Medical Research, Melbourne, Victoria, 3165, Australia; The Ritchie Centre, Hudson Institute of Medical Research, Melbourne, Victoria, 3165, Australia; Department of Women’s and Children’s Health, Astrid Lindgren Children’s Hospital, Karolinska Instituet, Stockholm, 17177, Sweden; The Ritchie Centre, Hudson Institute of Medical Research, Melbourne, Victoria, 3165, Australia; Department of Obstetrics and Gynaecology, Monash University, Melbourne, Victoria, 3168, Australia; The Ritchie Centre, Hudson Institute of Medical Research, Melbourne, Victoria, 3165, Australia; Department of Obstetrics and Gynaecology, Monash University, Melbourne, Victoria, 3168, Australia; The Ritchie Centre, Hudson Institute of Medical Research, Melbourne, Victoria, 3165, Australia; Department of Obstetrics and Gynaecology, Monash University, Melbourne, Victoria, 3168, Australia; The Ritchie Centre, Hudson Institute of Medical Research, Melbourne, Victoria, 3165, Australia; Department of Obstetrics and Gynaecology, Monash University, Melbourne, Victoria, 3168, Australia; The Ritchie Centre, Hudson Institute of Medical Research, Melbourne, Victoria, 3165, Australia; Department of Paediatrics, Monash University, Melbourne, Victoria, 3168, Australia

**Keywords:** preterm birth, mechanical ventilation, intrauterine inflammation, neuroinflammation, cardiorespiratory centres

## Abstract

Preterm newborns exposed to infection/inflammation *in utero* are at an increased risk of requiring respiratory support at birth, often in the form of mechanical ventilation. Mechanical ventilation and intrauterine inflammation independently cause inflammation in brainstem respiratory-related centres. However, the synergistic effect of intrauterine inflammation and mechanical ventilation on brainstem inflammation is unknown. We hypothesized that 24 h of mechanical ventilation after intratracheal LPS exposure exacerbates inflammation in the brainstem respiratory-related centres of preterm foetal sheep. Preterm foetal sheep (110 ± 1 days’ gestation) were surgically instrumented with catheters and tubing for *in utero* ventilation (VENT). At 115 ± 3 days, foetal sheep were randomly assigned to: (i) unventilated control + intratracheal (i.t.) saline (CONT_SAL_; *n* = 6), (ii) 24 h of VENT with i.t. saline (VENT_SAL_; *n* = 7), (iii) unventilated control with i.t. LPS (CONT_LPS_; *n* = 7) or (iv) 24 h VENT with i.t. LPS (VENT_LPS_; *n* = 6). *In utero* ventilation was started 1 h after i.t. LPS/saline administration, targeting a tidal volume of 3–5 mL/kg for 24 h. Serial plasma samples and post-mortem CSF were assessed for systemic and central inflammation, respectively. At 24 h, brainstem tissue was collected for molecular and histological assessment of markers of inflammation and injury. Plasma interleukin (IL)-1β, IL-6, IL-10 and interferon-γ-induced protein-10 (IP-10) were significantly increased in VENT_LPS_ foetuses compared to CONT_SAL_ and VENT_SAL_ (*P* < 0.05). IL-6 was higher in the cerebrospinal fluid of VENT_LPS_ groups compared to CONT_LPS_ (*P* = 0.0002). mRNA tumour necrosis factor (*TNF*) (*P* = 0.035) and prostaglandin-endoperoxide synthase-2 (*PTGS2*) (*P* = 0.011) were increased in the brainstems of VENT_LPS_ foetuses compared to CONT_LPS_. LPS exposure increased the number of astrocytes, microglia and STAT3+ cells within key respiratory-related centres compared to CONT_SAL_ and VENT_SAL_ (*P* < 0.01). Mechanical ventilation for 24 h after i.t. LPS amplifies markers of systemic and brainstem inflammation but does not further exacerbate histological inflammation or cell death in brainstem respiratory-related centres. The exacerbated inflammation suggests that mechanical ventilation preceded by intrauterine inflammation may impede cardiorespiratory control with adverse effects on spontaneous breathing and cardiovascular function in preterm infants.

## Teaser

Vidinopoulos *et al.* (2025) report that mechanical ventilation for 24 h preceded by intratracheal endotoxin exposure amplifies inflammatory proteins systemically and causes inflammation of brainstem respiratory-related centres in preterm foetal sheep. These changes may impede cardiorespiratory control with adverse effects on spontaneous breathing in preterm infants.

## Graphical Abstract

**Graphical Abstract:**
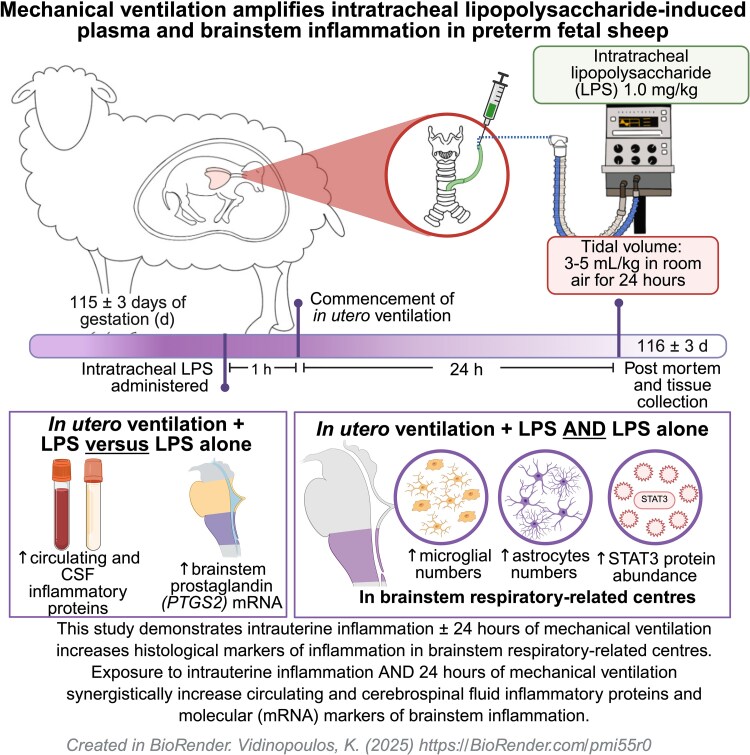

## Introduction

The brainstem is a specialized region of the central nervous system (CNS) that is responsible for the neural control of breathing. The brainstem contains discrete clusters of neurons in respiration-related centres that form a complex network that allows for the robust generation of rhythmic breathing, whilst maintaining flexibility to respond to physiological challenges and environmental stressors.^1-4^ The neural networks that control breathing develop during gestation and play a critical role in the respiratory adaptations that are required for a successful foetal to neonatal transition.^5,6^ Development of the neural architecture that is required for spontaneous breathing of the foetus is known to be compromised by prematurity and inflammation.^7,8^

Preterm birth (<37 weeks’ gestation) is associated with an increased risk of adverse neurodevelopmental outcomes,^9,10^ including but not limited to cerebral palsy.^11^ Furthermore, prematurity compromises the development of the ventilatory control network, resulting in an increased incidence of apnoeas and respiratory dysrhythmia at birth.^12^ This respiratory dysfunction increases the requirement for and duration of, respiratory support.^13^ Every hour of respiratory support increases the risk of brain injuries such as intraventricular haemorrhage and periventricular leukomalacia.^14^ Moreover, the number of days exposed to mechanical ventilation is associated with reduced volumes of the pons and medulla in preterm infants, suggesting that prolonged mechanical ventilation promotes structural remodelling in the brainstem.^15^ We have recently demonstrated the impact of mechanical ventilation on the brainstem respiratory-related centres in preterm foetal sheep, where 24 h of low tidal volume mechanical ventilation increased plasma pro-inflammatory cytokine expression and promoted a mild to moderate increase in histological markers of inflammation in brainstem respiratory-related centres, including the pre-Bötzinger complex (pre-BÖTC) and the retrotrapezoid nucleus and parafacial respiratory group (RTN/pFRG). The pre-BÖTC and RTN/pFRG are responsible for generating respiratory rhythm/inspiration and expiration, respectively.^16^ Indeed, inflammation within the brainstem respiratory-related centres may induce potential life-threatening dysregulation of respiratory drive in preterm infants and inhibit reflex responses to common environmental stressors faced during neonatal intensive care.^17-19^

Intrauterine infection/inflammation is a common antecedent of preterm birth.^20-22^ Importantly, exposure to intrauterine infection/inflammation and the resultant foetal pulmonary and systemic inflammatory response is mediated by the lungs.^23^ Human cohort and large animal studies have shown that intrauterine infection/inflammation is associated with brainstem inflammation and poor respiratory outcomes including the increased requirement and duration of respiratory support.^17,24,25^ Studies in neonatal mice have shown significant increases in markers of inflammation within brainstem respiratory-related centres that were associated with increased spontaneous apnoeas, duration of apnoeas and inability to autoresuscitate following respiratory challenge, including apnoea following intraperitoneal injection of IL-1β.^26,27^ Moreover, intratracheal lipopolysaccharide (LPS) exposure in premature mouse pups resulted in the upregulation of *IL1B* and *IL6* mRNA and depressed inhibitory signalling in the pre-BÖTC, inducing apnoea.^28,29^

Whilst it is well established that exposure to intrauterine infection/inflammation and extensive durations of respiratory support can independently result in brainstem inflammation and injury,^16-19,28^ the consequences of a combination of both pro-inflammatory and potentially injurious stimuli on brainstem respiratory-related centre pathology are not well established. We aimed to test the hypothesis that 24 h of mechanical ventilation following foetal lung inflammation induced by intratracheal LPS exposure will exacerbate brainstem inflammation and injury compared to either insult in isolation. To test this hypothesis, we used an established model of ventilation-induced lung and brain inflammation and injury, known as *in utero* ventilation (IUV).^30-32^ IUV induces a systemic inflammatory response in the absence of potential confounders of brainstem inflammation and injury, including haemodynamic instability and ionotropic support, associated with the delivery and intensive care that preterm sheep at this gestation would necessitate *ex utero*.^16,33^ Moreover, in this study, LPS was delivered intratracheally to replicate the entry of inflammatory stimuli (e.g. invading microorganisms and inflammatory proteins) from the amniotic space into the foetal circulation via the lungs, demonstrated to be a key pathway in initiating the foetal inflammatory response.^23^

## Materials and methods

### Ethics approval

The Hudson Institute of Medical Research Animal Ethics Committee [approval number MMCA (Monash Medical Centre Animal Ethics Committee A) 2020/15] approved all procedures. This study was conducted in accordance with the local legislation and institutional requirements and also with the ARRIVE (Animal Research: Reporting In Vivo Experiments) guidelines 2.0^34^ and the National Health and Medical Research Council Code of Practice for the Care and Use of Animals for Scientific Purposes (Eighth Edition).

### Foetal surgery

Twenty-six Border Leicester ewes carrying singleton or twin foetuses underwent aseptic surgery at 110 ± 1 days gestational age (term = 147 days). Foetuses were randomly assigned to one of four groups:

1. Un-ventilated controls + saline (CONT_SAL_; *n* = 6)
2. *In-utero* ventilation + saline (VENT_SAL_; *n* = 7)
3. Unventilated controls + LPS (CONT_LPS_; *n* = 7)
4. *In-utero* ventilation + LPS (VENT_LPS_; *n* = 6).

### Foetal instrumentation

Foetal instrumentation was performed under sterile conditions as previously described.^16,35,36^ Briefly, food but not water was withheld ∼18 h prior to instrumentation surgery. Ewes were anaesthetized with intravenous (i.v.) sodium thiopentone (20 mg/kg) and anaesthesia was maintained with 2–3% isoflurane in oxygen via a positive pressure ventilator. Prophylactic antibiotics were administered to the ewe (ampicillin, 1 g i.v. and engemycin, 500 mg i.v.). The foetal head and the left forelimb were exteriorized via a midline maternal laparotomy for instrumentation. Foetuses randomized to VENT_SAL_ and VENT_LPS_ groups underwent a tracheostomy procedure to instrument each foetus with a ventilation circuit as previously described.^16,35,36^ Foetuses randomized to CONT_SAL_ and CONT_LPS_ also underwent a tracheostomy for the insertion of a single non-occlusive tracheal catheter (I.D. 3.46 mm, O.D. 8.6 mm). Heparinized polyvinyl catheters were inserted into the left brachial artery, left jugular vein and amniotic sac. Postoperative analgesia and antibiotics were maintained for 3 consecutive days. Analgesia was maintained via a transdermal fentanyl patch placed on the left hind leg of the ewe (75 g/h) and antibiotics administered i.v. to the ewe (ampicillin, 1 g and engemycin, 500 mg) and the foetus (ampicillin, 200 mg).

### IUV initiation and LPS administration

Experimental procedures for all groups μat 115 ± 3 days’ gestation. Foetuses first received intratracheal LPS (*Escherichia coli*, 055:B5, Millipore Sigma, MO, USA; 1 mg in 2 mL saline), or saline (vehicle, 2 mL), either via the ventilation circuit (VENT_LPS_ and VENT_SAL_) or the tracheal catheter (CONT_LPS_ and CONT_SAL_). The tracheal loop of VENT_SAL_ and VENT_LPS_ was cut, and lung liquid was passively drained, recorded and discarded before LPS or saline was administered intratracheally. Ventilation was initiated *in utero* in VENT_LPS_ and VENT_SAL_ foetuses 1 h after LPS administration. The tracheal tube was connected to a neonatal ventilator (Drager 8000+, Lübeck, Germany), and ventilation was provided in pressure support mode with a maximum peak inflation pressure (PIP) of 45 cmH_2_O and a positive end-expiratory pressure of 5 cmH_2_O. PIP were adjusted to achieve a target tidal volume (*V*_T_) of 3–5 mL/kg, sufficient to achieve normocapnia at this stage of lung maturity.^37^ An inspiratory flow of 10 L/min, respiratory rate of 60 breaths/min and fraction of inspired oxygen (FiO_2_) of 0.21 were used. Foetuses were continuously ventilated for a total duration of 24 h with non-humidified gas. Foetal arterial blood and plasma samples were collected at baseline (pre-LPS), 1 h following LPS (pre-ventilation) and at the onset of ventilation (or equivalent for CONT_SAL_ and CONT_LPS_ groups), at 15, 30 and 45 min and at 1, 3, 6, 9, 12 and 24 h after ventilation onset for analysis of arterial blood PaO_2_, PaCO_2_, pH, SaO_2_, glucose and lactate concentrations (ABL90 Flex Plus analyser, Radiometer, Brønshøj, Denmark) and plasma collection for cytokine analysis (Supplementary Fig. 1). 24 h later (or equivalent for CONT_SAL_ and CONT_LPS_ groups), ewes and foetuses were humanely euthanized via sodium pentobarbitone overdose (100 mg/kg i.v., Virbac, NSW, Australia).

### Cerebrospinal fluid and tissue collection

At post-mortem, cerebrospinal fluid (CSF) was collected via puncture of the cisternae magna. CSF was centrifuged, and the supernatant was aliquoted and stored at −80°C. The brainstem was isolated between the peduncles and thalamus, then bisected in the sagittal plane. The left brainstem was frozen in liquid nitrogen, while the right side was dissected into the pons and medulla and immersion-fixed in 10% phosphate-buffered formalin for 7 days at 4°C before embedding and sectioning as described previously.^16,38^ Respiratory-related centres of the brainstem included the pre-B$\ddot{O}$TC, RTN/pFRG, the nucleus tractus solitarius (NTS), the nucleus ambiguus (NA) and the raphe nucleus (RN) (Supplementary Fig. 2).

### Plasma and CSF protein concentrations

Arterial plasma samples collected at baseline, 1, 3, 6, 12 and 24 h, and CSF samples collected at post-mortem were assessed for cytokine concentrations. Interleukin (IL)-1*β*, IL-6, IL-8, IL-10, tumour necrosis factor (TNF) and interferon-*γ*-induced protein (IP-10) were measured using Milliplex MAP bovine cytokine magnetic bead panel assay kits (cat#: BCYT1-33 K; Merck Millipore, Burlington, MA, USA). Sampling timepoints were selected based on a previous study employing a similar experimental paradigm.^16^ Protein concentrations of IL-1*β*, IL-6, IL-8, IL-10, TNF and IP-10 were quantified using a Bio-Plex MAGPIX® Multiplex reader with xPOTENT® software (Bio-Rad, CA, USA). Each assay included internal quality controls, and cytokine levels were within the lower detection limit across all samples. Samples that reached the upper detection limit of the assay (12 000 pg/mL) have been reported as = 12 000 pg/mL. One CSF sample from VENT_SAL_ was not collected.

### Brainstem gene analysis

RNA extraction, cDNA preparation and gene expression analysis were conducted as previously described.^16^ Briefly, the left side of the brainstem (100–150 mg) was homogenized and mRNA extracted using the RNeasy Midi RNA extraction kit (Qiagen, Venlo, Netherlands) according to the manufacturer’s instructions. Extracted RNA was reverse transcribed into single-stranded cDNA (Superscript III First-strand synthesis, Invitrogen, MA, USA). High-throughput real-time quantitative polymerase chain reaction (RT-qPCR) was performed with microfluidic technology, Fluidigm Access Array System (Fluidigm Corporation, CA, USA). Genes of interest included inflammatory cytokines *IL1A*, *IL1B*, *IL6*, *IL8*, *IL10*, *IL18*, *TNF*; markers of inflammation including high mobility group box 1 (*HMGB1*), nuclear factor kappa B (*NFκB*), CXC motif chemokine ligand 10 (*CXCL10*), *FOXP3*, myeloperoxidase (*MPO*), matrix metallopeptidase 9 (*MMP9*), toll-like receptor (*TLR*)*4*, serum amyloid A (SAA); markers of cell death/damage including heat shock protein-70 (*HSP70*), caspase (*CASP*)*1*, *CASP3*, *CASP8*; markers of prostaglandin synthesis prostaglandin E synthase (*PTGES*) and prostaglandin-endoperoxide synthase-2 (*PTGS2*). Details of TaqMan gene array ovine-specific probes used for PCR can be found in Supplementary Table 1. Using SYBR chemistry, quality control testing was performed for the housekeeping genes *18S*, *B2M* and *S29*. The geomean of the three housekeeping genes was calculated, and the value was used to determine the normalized expression of each gene of interest.

### Immunofluorescence and immunohistochemistry

Double labelling of astrocytes [mouse anti-glial fibrillary acidic protein (GFAP); 1:500, Sigma, cat#. G3893], microglia [rabbit anti-ionized calcium binding adaptor molecule (IBA1), 1:500, Wako, 019-19,741], STAT3 [rabbit anti phospho-STAT3 protein; 1:200, Cell Signalling, cat#9145] and NeuN [mouse anti-NeuN antibody, 1:200, Abcam, #ab279296]-positive cells were performed using immunofluorescence. Sections were incubated in either 1:200 goat anti-mouse Alexa Fluor 594 (cat#: 115-585-003, Jackson ImmunoResearch, West Grove, PA, USA) and 1:200 goat-anti-rabbit- Alexa Fluor 488 (cat#: 111-545-144, Jackson ImmunoResearch) secondary antibodies in 2% NGS and 0.1% PBST for 2 h at room temperature. Nuclei staining was performed with HOECHST 33342 trihydrochloride, trihydrate 1:1000 (Cat#: H3570, Invitrogen, ThermoFisher Scientific). DAKO anti-fade mounting medium (cat#: GM30411-2, Agilent Technologies, CA, USA) was used to coverslip slides. Terminal deoxynucleotidyl transferase dUTP nick end labelling (TUNEL) was performed using ApopTag® Peroxidase Kit to identify cells undergoing *in-situ* apoptosis, as per the manufacturer’s instructions (Millipore S7100; CA, USA).

### Histology quantification

Sections were imaged at 20× magnification using QuPath imaging software (Version 0.2.3).^39^ Four non-overlapping and random fields of view (width: 319.95 μm; length: 407.19 μm; area: 2.3 mm^2^) were taken from each section. Numbers of positively stained cells or immunoreactivity (% area fraction of staining) were quantified from 2 sections per brainstem respiratory-related centre of interest from each subject using either ImageJ imaging software or QuPath imaging software (v2.00, LOCI, University of Wisconsin). A single observer (K.V.), blinded to the treatment of each group, coded and assessed all sections. GFAP+ astrocytes were quantified by identifying astrocyte cell bodies and expressed as GFAP+ cells per field. The % area fraction/coverage per field of GFAP+ astrocytes was also measured as hypertrophy of the processes of astrocytes is a common adaptation in response to pathology within the CNS.^40^ Numbers of microglia (IBA1+ cells) were quantified as total IBA1+ cells and then further classified according to their activated morphology/phenotypically amoeboid microglia (large cell bodies, with ≤1 branching process).^41,42^ STAT3 positive cells were quantified according to double labelling with nuclear marker HOESCHT and expressed per field of view (STAT3+ cells/field). ApopTag (TUNEL)+ cells were quantified as total numbers of immunopositive cells per field of view (TUNEL+ cells/field). Data are presented for each brainstem respiratory-related centre of interest.

### Statistical analysis

Data were analysed using GraphPad Prism Software. A Shapiro–Wilk test was first used to assess for normality. Data violating sphericity were corrected with the Greenhouse–Geisser method. Arterial blood gas parameters and circulating plasma proteins were analysed by mixed effects to assess the main effects of ventilation (*P*_VENT_), LPS (*P*_LPS_) and time (*P*_TIME_). Where a significant interaction between variables was found, Sidak’s or Tukey’s *post hoc* tests were utilized where appropriate. To account for baseline differences between groups, circulating plasma proteins were expressed as fold change from baseline. Animal characteristics, qPCR, CSF protein concentrations and immunohistochemical data sets were assessed for the main effects of ventilation (*P*_VENT_), LPS (*P*_LPS_), and interactions between ventilation and LPS (*P*_VENT X LPS_) by two-way ANOVA. Tukey’s *post hoc* test was used to determine differences where significant interactions were observed. Area under the curve (AUC) was calculated for circulating plasma proteins using GraphPad Prism and assessed for main effects of ventilation (*P*_VENT_), LPS (*P*_LPS_), and interactions between ventilation and LPS (*P*_VENT X LPS_) using two-way ANOVA with Tukey’s multiple comparisons as a *post hoc* test. Linear regressions were used to compare the relationship between plasma inflammatory proteins and brainstem respiratory-related centres, where higher numbers of microglia, astrocytes and STAT3+ cells were observed, including the pre-B$\ddot{O}$TC and RTN/pFRG. Power analysis indicated that the study had 80% power to detect a 25% increase in GFAP+ cell numbers between groups at an alpha level of 0.05. Statistical significance was defined as *P* < 0.05. Data are mean ± standard error of the mean (SEM).

## Results

### Foetal characteristics

Foetal characteristics at post-mortem and baseline arterial blood gas parameters are presented in Supplementary Table 2. Foetal body weight (kg), sex (%male), brain weights (g) and rate of twin pregnancies (%) were not different between groups. Volume of lung liquid drained was not different between VENT_SAL_ (117 ± 33 mL) and VENT_LPS_ (130 ± 21 mL) groups (*P* = 0.28). Foetuses randomized to VENT_SAL_ and VENT_LPS_ groups received a mean tidal volume of 2.8 mL/kg (range 2.5–3.1 mL/kg). One sample, randomized to VENT_SAL_, was ventilated for 24.5 h.

### Foetal biochemistry

Foetal arterial blood biochemistry obtained at baseline (pre-LPS) and throughout the 24 h experimental period is presented in Supplementary Fig. 3. Arterial blood pH (*P*_TIME X VENT_ = 0.015, Supplementary Fig. 3A) and arterial partial pressure of oxygen (PaO_2_) (*P*_TIME X VENT_ < 0.0001, Supplementary Fig. 3B) differences were mediated by ventilation (VENT_SAL_ and VENT_LPS_) exposure. The partial pressure of carbon dioxide (PaCO_2_) (*P*_TIME X LPS_ = 0.011) was higher in LPS-exposed (CONT_LPS_ and VENT_LPS_) foetuses at 30 min (*P* = 0.04) and between 3 and 9 h (*P* < 0.05, Supplementary Fig. 3C) of ventilation. Oxygen saturation (SaO_2_) was lower in VENT_LPS_ compared to CONT_SAL_ and VENT_SAL_ at 15 min and between 3 and 24 h (*P* < 0.05, Supplementary Fig. 3D). At 12 h, SaO_2_ was lower in VENT_LPS_ compared to CONT_LPS_ (*P* = 0.021, Supplementary Fig. 3D). Changes in glucose concentration (cGlu) were mediated by LPS exposure (*P*_TIME X LPS_ = 0.046, CONT_LPS_ and VENT_LPS_, Supplementary Fig. 3E). Lactate concentrations (cLac) were higher in VENT_LPS_ compared to CONT_SAL_ and VENT_SAL_ at 60 min, 3, 6 and 9 h of ventilation (*P* < 0.05; Supplementary Fig. 3F). cLac was higher in CONT_LPS_ compared to VENT_SAL_ and CONT_SAL_ between 3 and 9 h (*P* < 0.05). cLac was higher in CONT_LPS_ compared to CONT_SAL_ at 12 h (*P* = 0.034).

### Circulating inflammatory markers

**Figure 1:**
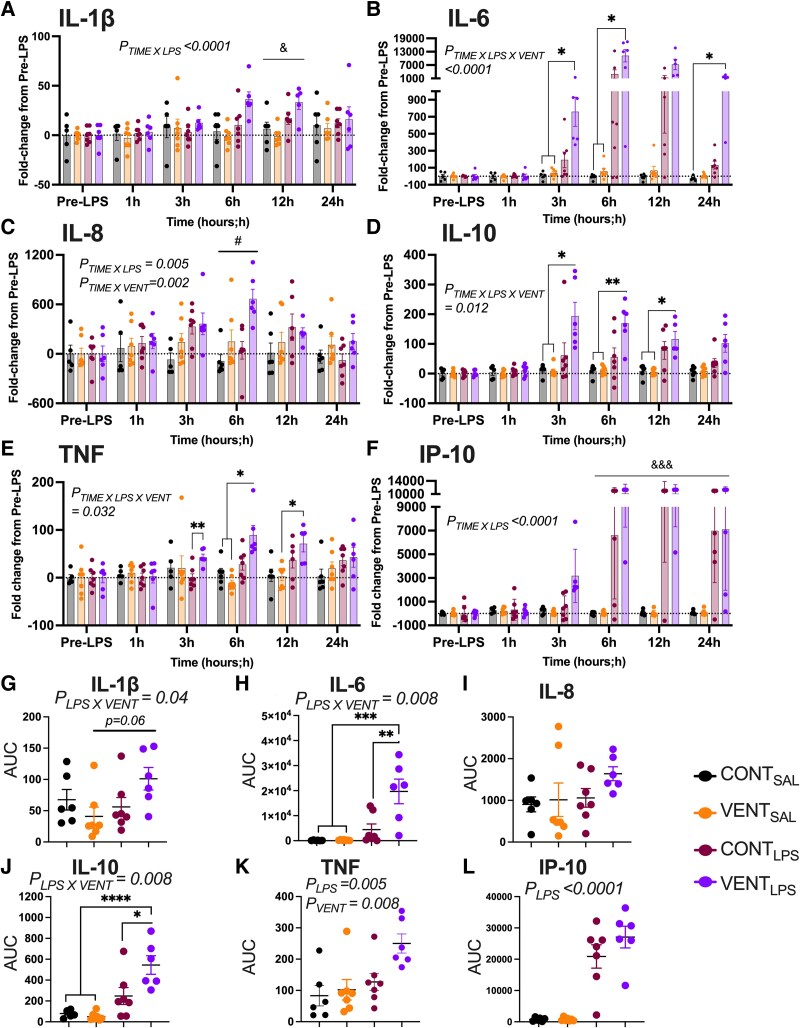
**Plasma inflammatory proteins.** Plasma (**A**) interleukin (IL)-1β, (**B**) IL-6, (**C**) IL-8, (**D**) IL-10, (**E**) tumour necrosis factor (TNF) and (**F**) interferon protein (IP)-10 taken from baseline (pre-LPS) and throughout the 24 h of ventilation from control (CONT_SAL_; black circles; *n* = 6), ventilation + saline (VENT_SAL_; orange circles; *n* = 7), un-ventilated controls + LPS (CONT_LPS_; burgundy circles; *n* = 7) and ventilation + LPS (VENT_LPS_; purple circles; *n* = 6). Each data point represents a single animal (**A–F**). Presented as fold-change from pre-LPS. Data are mean ± SEM. Mixed effects with Tukey’s *post hoc* differences indicated as *P < 0.05 and **P < 0.01. Two-way ANOVA with Sidak’s *post hoc* differences between CONT v LPS indicated as ^&^*P* < 0.05 and ^&&&^*P* < 0.001, CONT v VENT indicated as ^#^*P* < 0.05. AUC (cumulative) calculations for (**G**) IL-1β, (**H**) IL-6, (**I**) IL-8, (**J**) IL-10, (**K**) TNF and (**L**) IP-10 from CONT_SAL_, VENT_SAL_, CONT_LPS_ and VENT_LPS_. Two-way ANOVA with Tukey’s *post hoc* was used to calculate differences indicated as *P < 0.05, **P < 0.01, ***P < 0.001, ****P < 0.0001. Each data point represents the AUC for each foetus (**G–L**).

Circulating inflammatory proteins are presented as fold change from pre-LPS. IL-1β fold change was higher in LPS-exposed foetuses (*P*_TIME X LPS_ < 0.0001, CONT_LPS_ and VENT_LPS_, Fig. 1A) at 12 h. IL-6 fold change was higher in VENT_LPS_ foetuses compared to CONT_SAL_ and VENT_SAL_ (*P* < 0.04) between 3 and 6 h of ventilation (Fig. 1B). IL-8 fold change was higher in ventilation (*P*_TIME X VENT_ = 0.002, VENT_SAL_ and VENT_LPS_) exposed groups at 6 h (Fig. 1C). IL-10 fold change was higher in VENT_LPS_ compared to CONT_SAL_ and VENT_SAL_ between 3 and 12 h (*P* < 0.05, Fig. 1D). Fold change TNF was higher in VENT_LPS_ compared to CONT_LPS_ (*P* < 0.001) at 3 h, CONT_SAL_ at 6 h (*P* = 0.003) and VENT_SAL_ between 6 and 12 h (*P* < 0.05, Fig. 1E). IP-10 fold change was higher in LPS (*P*_TIME X LPS_ < 0.0001, CONT_LPS_ and VENT_LPS_) exposed groups between 6 and 24 h (Fig. 1F). Cumulative plasma IL-1β, as measured by AUC, was not different between groups (*P* = 0.06, Fig. 1G). Cumulative IL-6 concentration was highest in VENT_LPS_ compared to CONT_SAL_ (*P* < 0.001), VENT_SAL_ (*P* < 0.0001) and CONT_LPS_ (*P* = 0.0004) foetuses (Fig. 1H), but no differences were found in cumulative IL-8 (Fig. 1I). Cumulative IL-10 was highest in VENT_LPS_ compared to CONT_SAL_ (*P* < 0.0001), VENT_SAL_ (*P* < 0.0001) and CONT_LPS_ (*P* < 0.002, Fig. 1J) but no differences were found in cumulative TNF (Fig. 1K). Cumulative IP-10 was higher in LPS-exposed foetuses, including CONT_LPS_ and VENT_LPS_ (*P*_LPS_ < 0.0001, Fig. 1L).

### CSF inflammatory proteins

**Figure 2:**
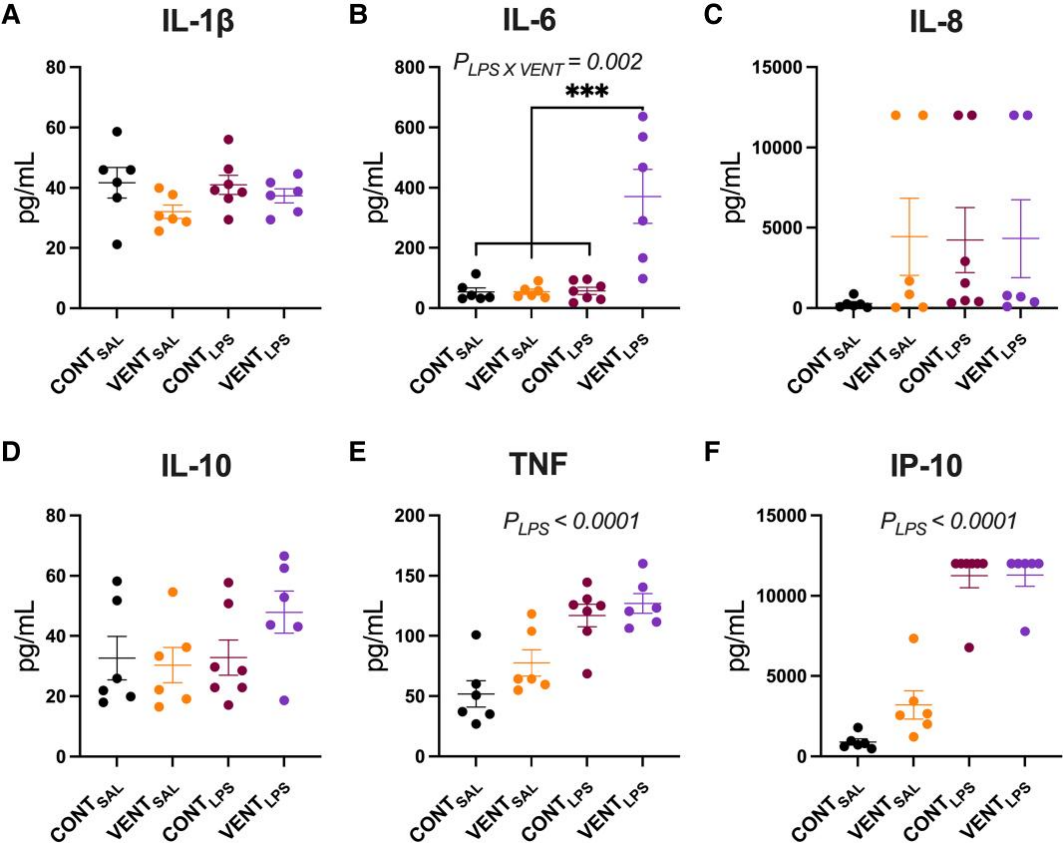
**CSF inflammatory proteins.** CSF protein concentrations (pg/mL) of (**A**) interleukin (IL)-1β, (**B**) IL-6, (**C**) IL-10, (**D**) IL-10, (**E**) tumour necrosis factor (TNF) and (**F**) interferon protein (IP)-10 from control (CONT_SAL_; *n* = 6), ventilation + saline (VENT_SAL_; *n* = 6, un-ventilated controls + LPS (CONT_LPS_; burgundy circles; *n* = 7) and ventilation + LPS (VENT_LPS_; *n* = 6). Concentrations above the upper detection limit of the assay (>12 000 pg/mL) are reported as =12 000 pg/mL. Data are mean ± SEM. Two-way ANOVA with Tukey’s *post hoc*. ***P < 0.001. Each data point represents the CSF concentration for each protein for each foetus.

Protein concentrations of cytokines in the CSF at post-mortem of LPS and ventilation-exposed foetal sheep are shown in Fig. 2. Protein concentration of CSF IL-1β was not different between groups (Fig. 2A). CSF IL-6 protein concentration was higher in VENT_LPS_ foetuses compared to CONT_LPS_ (*P* = 0.0002), VENT_SAL_ (*P* = 0.0003) and CONT_SAL_ (*P* = 0.0003, Fig. 2B). IL-8 (Fig. 2C) and IL-10 (Fig. 2D) protein concentrations in the CSF were not different between groups. TNF (Fig. 2E) and IP-10 (Fig. 2F) protein concentrations were highest in LPS-exposed foetuses (*P*_LPS_ < 0.0001, CONT_LPS_ and VENT_LPS_).

### Brainstem mRNA expression

**Figure 3:**
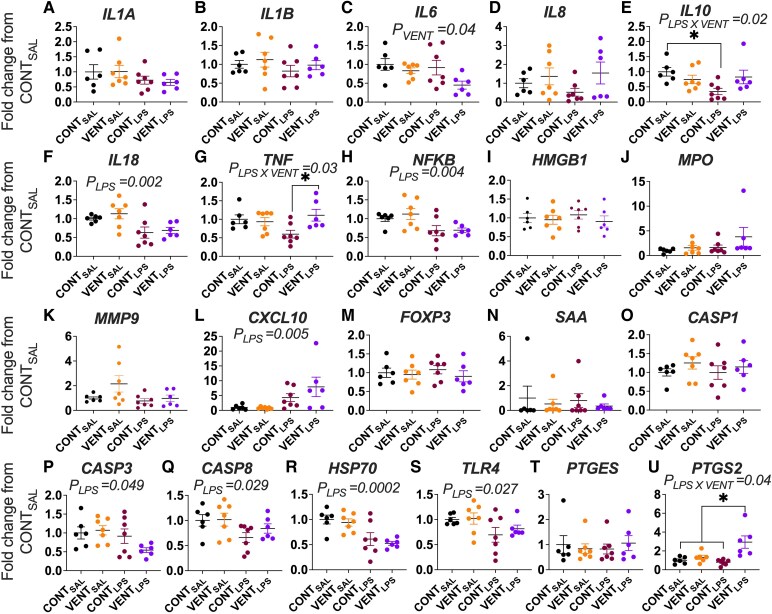
**Brainstem mRNA expression.** mRNA expression of (**A**) interleukin (*IL)1A*, (**B**) *IL1B*, (**C**) *IL6*, (**D**) *IL8*, (**E**) *IL10*, (**F**) *IL18*, (**G**) tumour necrosis factor (*TNF*), (**H**) nuclear factor kappa B (*NFκB*), (**I**) high mobility group box 1 (*HMGB1*), (**J**) myeloperoxidase (*MPO*), (**K**) matrix metallopeptidase (*MMP9*), (**L**) CXC motif chemokine ligand 10 (*CXCL10*), (**M**) *FOXP3*, (**N**) serum amyloid A (*SAA*), (**O**) caspase (*CASP*)*1*, (**P**) *CASP3*, (**Q**) *CASP8*, (**R**) heat shock protein 70 (*HSP70*), (**S**) toll-like receptor (*TLR*)*4*, (**T**) prostaglandin E synthase (*PTGES*) and (**U**) prostaglandin-endoperoxidase synthase-2 (*PTGS2*) in control (CONT_SAL_; *n* = 6), ventilation + saline (VENT_SAL_; *n* = 7), un-ventilated controls + LPS (CONT_LPS_; *n* = 7) and ventilation and + LPS (VENT_LPS_; *n* = 6) presented as fold change from CONT_SAL_. Data are mean ± SEM. Two-way ANOVA with Tukey’s *post hoc*. *P < 0.05. Each data point represents the fold-change calculation for each gene for each individual foetus.

Brainstem mRNA expression of pro- and anti-inflammatory cytokines, inflammatory markers, cell damage/death markers and markers of prostaglandin synthesis are shown in Fig. 3. The mRNA expression of *IL1A* (Fig. 3A) and *IL1B* (Fig. 3B) was not different between groups. *IL6* mRNA expression was lower in ventilation-exposed groups (*P*_VENT_ = 0.04, VENT_SAL_ and VENT_LPS_, Fig. 3C). The mRNA expression of *IL8* (Fig. 3D) was not different between groups; however, the expression of *IL10* was lower in CONT_LPS_ compared to CONT_SAL_ (*P* = 0.033, Fig. 3E). *IL18* (*P*_LPS_ = 0.002, Fig. 3F) and *NFKB* (*P*_LPS_ = 0.004, Fig. 3H) mRNA were lower in LPS-exposed groups (CONT_LPS_ and VENT_LPS_) while *TNF* was higher in VENT_LPS_ compared to CONT_LPS_ (*P* = 0.035, Fig. 3G). The mRNA expression of *HMBG1*, *MPO* and *MMP9* was not different between groups (Fig. 3I–K). *CXCL10* mRNA expression was higher in LPS-exposed groups (*P*_LPS_ = 0.005, CONT_LPS_ and VENT_LPS_, Fig. 3L). The mRNA expression of *FOXP3*, *SAA* and *CASP1* was not different between groups (Fig. 3M–O). *CASP3* (*P*_LPS_ = 0.049), *CASP8* (*P*_LPS_ = 0.029), *HSP70* (*P*_LPS_ = 0.0002) and *TLR4* (*P*_LPS_ = 0.027) mRNA expression were lower in LPS-exposed groups (CONT_LPS_ and VENT_LPS_, Fig. 3P–S). *PTGES* mRNA expression was not different between groups (Fig. 3T), but *PTGS2* mRNA expression was highest in VENT_LPS_ compared to CONT_SAL_ (*P* = 0.004), VENT_SAL_ (*P* = 0.019) and CONT_LPS_ (*P* = 0.001, Fig. 3U).

### Medulla histopathology

**Figure 4:**
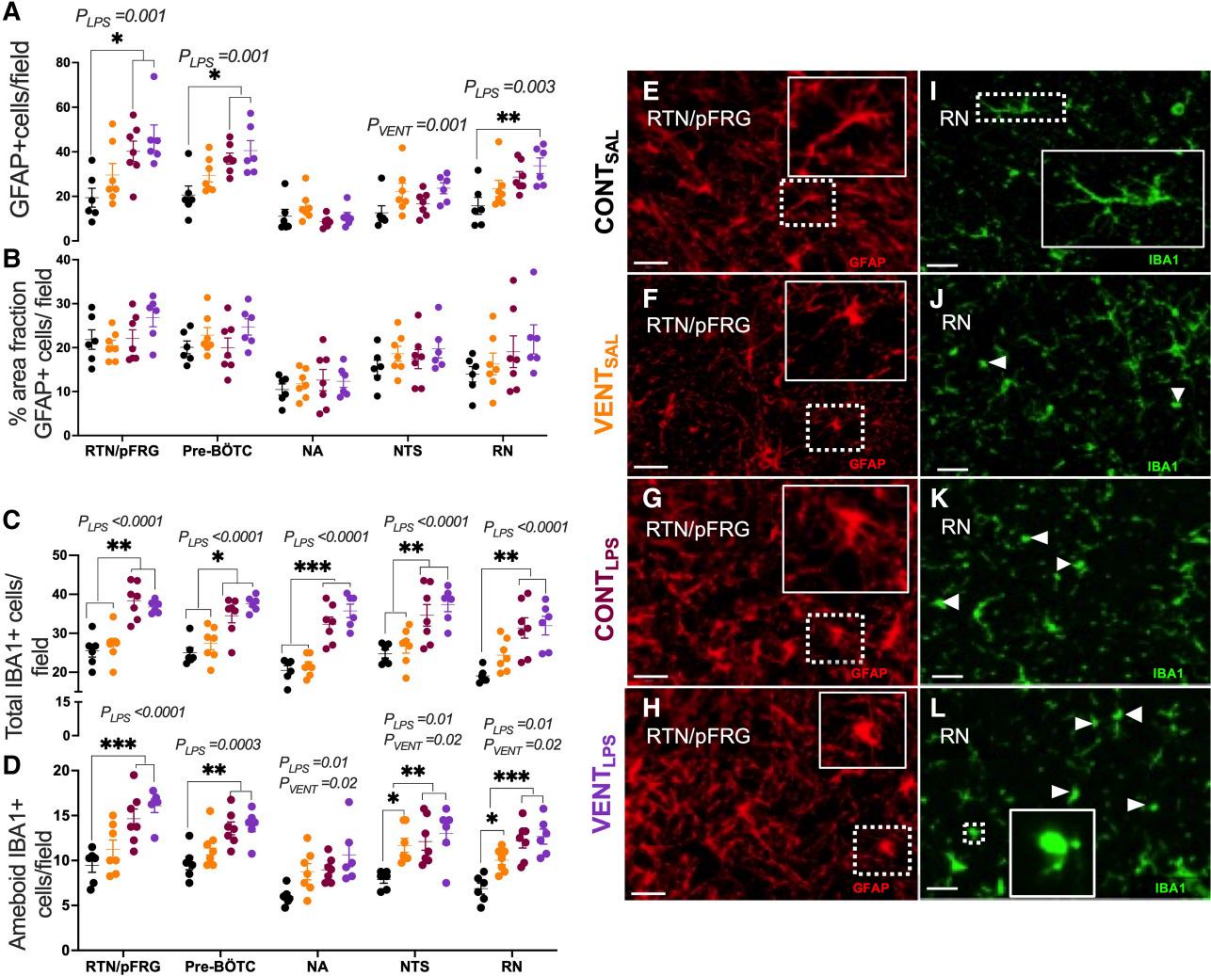
**Astrocyte and microglia expression in medullary respiration-related centres.** (**A**) Numbers of glial fibrillary acidic protein (GFAP)+ cells indicating astrocyte cell populations, (**B**) percentage area fraction of GFAP+ staining, (**C**) Total ionized binding adaptor molecule (IBA1+) per field and (**D**) amoeboid IBA1+ microglia per field in regional respiration-related centres including the RTN/pFRG, pre-BÖTC, nucleus ambiguus (NA), nucleus tractus solitarius (NTS) and the raphe nucleus (RN), in control (CONT_SAL_; black circles; *n* = 6), ventilation + saline (VENT_SAL_; orange circles; *n* = 7), un-ventilated controls + LPS (CONT_LPS_; burgundy circles; *n* = 7) and ventilation + LPS (VENT_LPS_; purple circles; *n* = 6). Data are mean ± SEM. Two-way ANOVA with Tukey’s *post hoc*. *P < 0.05, **P < 0.01, ***P < 0.001. Each data point represents each individual foetus. (**E–H**) Representative micrographs of GFAP (red) staining in the RTN/pFRG in (**E**) CONT_SAL_, (**F**) VENT_SAL_, (**G**) CONT_LPS_ and (**H**) VENT_LPS_ exposed foetuses. (**I–L**) Representative micrographs of IBA1 (green) staining in the raphe of (**I**) CONT_SAL_, (**J**) VENT_SAL_, (**K**) CONT_LPS_ and (**L**) VENT_LPS_ exposed foetuses. Scale = 20 μm. Inserts are zoomed images of the dashed boxes. White arrowhead indicates ameboid microglial phenotypes.

Numbers of GFAP+ astrocytes were higher in CONT_LPS_ (*P* = 0.033) and VENT_LPS_ (*P* = 0.010) foetuses compared to CONT_SAL_ in the RTN/pFRG and the pre-BÖTC (*P* = 0.015 and *P* = 0.010, respectively; Fig. 4A). GFAP+ astrocytes were higher in VENT_LPS_ compared to CONT_SAL_ in the RN (*P* = 0.008, Fig. 4A). The % area fraction of GFAP+ astrocytes was not different between groups (Fig. 4B). The number of IBA1+ cells was higher in all respiratory-related centres of interest following LPS-exposure (*P*_LPS_ < 0.01, Fig. 4C), except for the NTS, where VENT_LPS_ foetuses had a higher number of IBA1+ cells compared to CONT_SAL_ (*P* = 0.007) and VENT_SAL_ (*P* = 0.034, Fig. 4C). Morphological subclassification of IBA1+ cells found that numbers of amoeboid (activated) microglia were higher in VENT_LPS_ compared to CONT_SAL_ across all respiratory-related centres investigated (*P* < 0.01, Fig. 4D). CONT_LPS_ had a higher number of amoeboid microglia within the RTN/pFRG (*P* = 0.004), pre-BÖTC (*P* = 0.007), NTS (*P* = 0.015) and RN (*P* = 0.0001) compared to CONT_SAL_. The number of amoeboid microglia in VENT_SAL_ was higher in the NTS (*P* = 0.031) and RN (*P* = 0.020) compared to CONT_SAL_.

**Figure 5:**
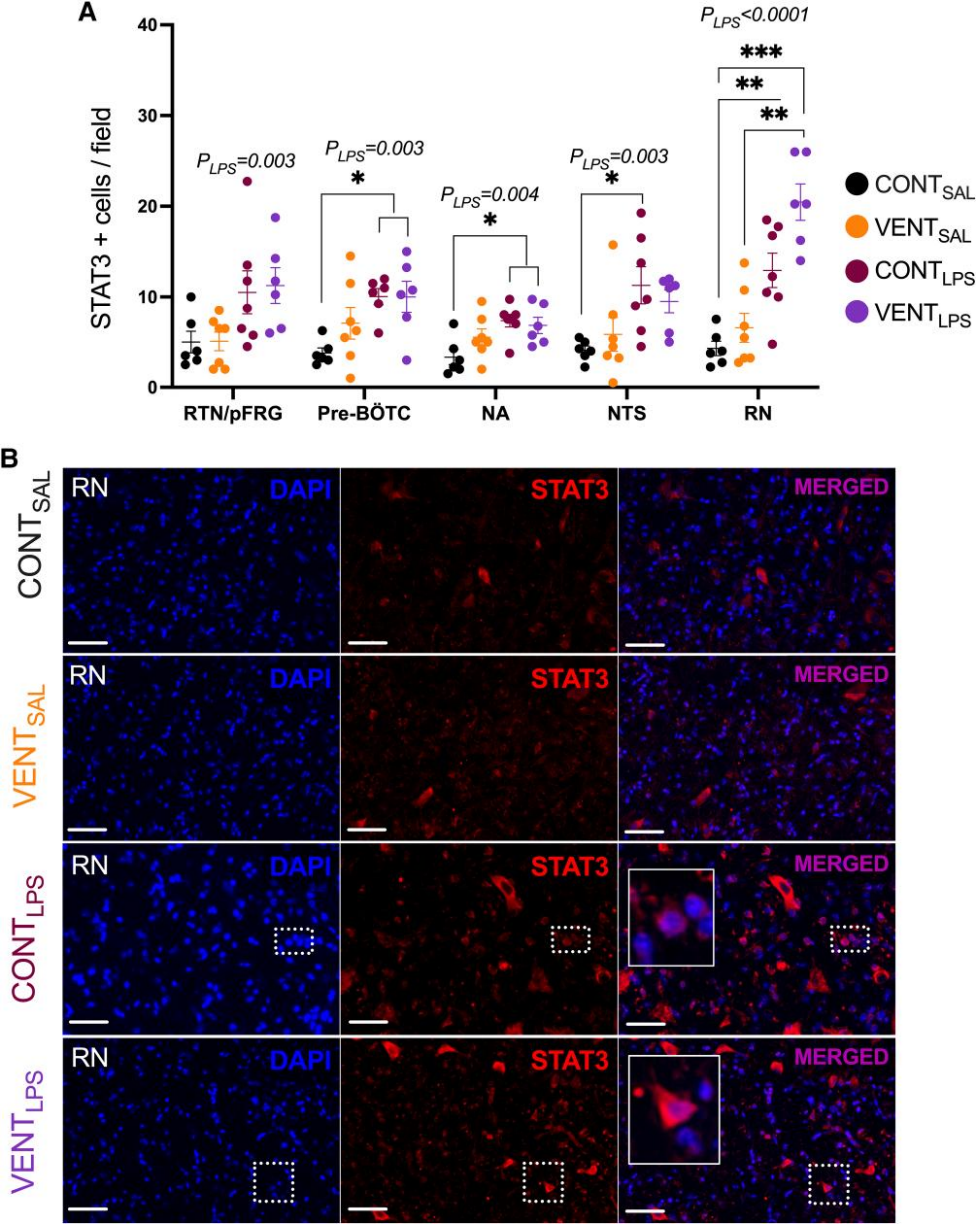
**STAT-3 + cells in medullary respiration-related centres.** (**A**) STAT-3 immunopositive cells per field of view in regional respiration-related centres including the RTN/pFRG, pre-BÖTC, nucleus ambiguus (NA), nucleus tractus solitarius (NTS) and the raphe nucleus (RN), in control (CONT_SAL_; black circles; *n* = 6), ventilation + saline (VENT_SAL_; orange circles; *n* = 7), un-ventilated controls + LPS (CONT_LPS_; burgundy circles; *n* = 7) and ventilation + LPS (VENT_LPS_; purple circles; *n* = 6). Data are mean ± SEM. *P < 0.05, **P < 0.01, ***P < 0.001. Each data point represents each individual foetus. (**B**) Representative micrographs of DAPI (blue), STAT-3 (red) and merged staining in the raphe of CONT_SAL_, VENT_SAL_, CONT_LPS_ and VENT_LPS_ exposed foetuses. Inserts are zoomed images of the dashed boxes. Scale = 20 μm.

**Figure 6:**
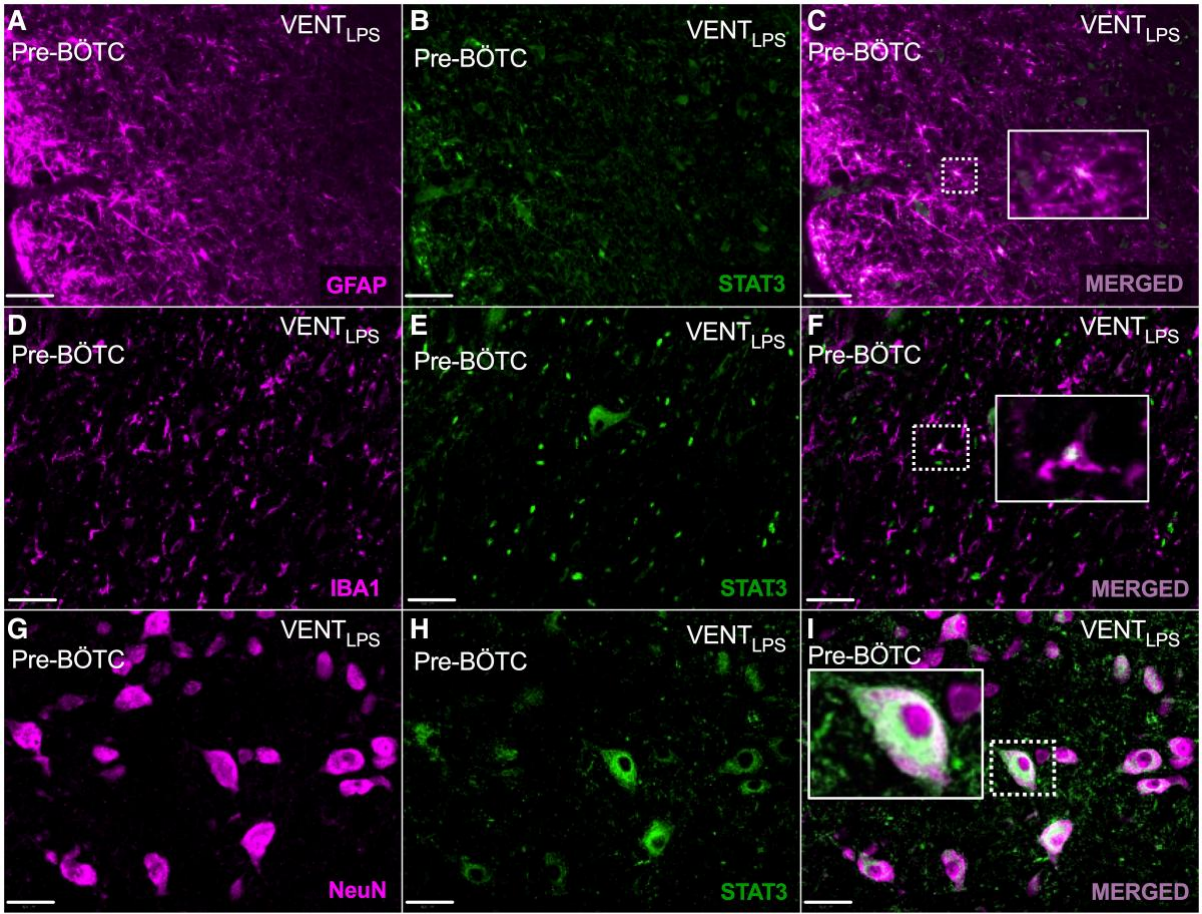
**STAT-3+ glial cells in the pre-BÖTC.** Representative photomicrographs showing positive double labelling of (**A**) glial fibrillary acidic protein (GFAP)+ astrocytes (magenta), (**D**) ionized binding adaptor (IBA1)+ microglia (magenta) and (**G**) NeuN+ neurons (magenta) with STAT-3+ cells (**B**, **E**, **H**) (green) and merged (**C**, **F**, **I**) in the pre-BÖTC of preterm foetal sheep exposed to 24 h of IUV following i.t. LPS administration (VENT_LPS_). Scale = 50 μm. Inserts are zoomed images of the dashed boxes.

The number of STAT3+ cells/field was higher in CONT_LPS_ compared to CONT_SAL_ foetuses in the pre-BÖTC (*P* = 0.011) and NTS (*P* = 0.024, Fig. 5A). The number of STAT3+ cells was higher in the NA of VENT_LPS_ (*P* = 0.042) and CONT_LPS_ (*P* = 0.013) compared to CONT_SAL_. There was a higher number of STAT3+ cells/field in the RN of VENT_LPS_ compared to VENT_SAL_ (*P* = 0.002) and CONT_SAL_ (*P* = 0.0003). The number of STAT3+ cells in the RN was higher in CONT_LPS_ (*P* = 0.009) and VENT_LPS_ (*P* = 0.0003) foetuses compared to CONT_SAL_. VENT_LPS_ foetuses also had higher STAT3+ cell numbers in the RN compared to VENT_SAL_ (*P* = 0.002). Qualitatively, STAT3+ staining was co-localized with GFAP+ astrocytes (Fig. 6A–C), IBA1+ microglia (Fig. 6D–F) and NeuN+ neurons (Fig. 6G and H) in the pre-BÖTC of VENT_LPS_-exposed foetuses.

**Figure 7:**
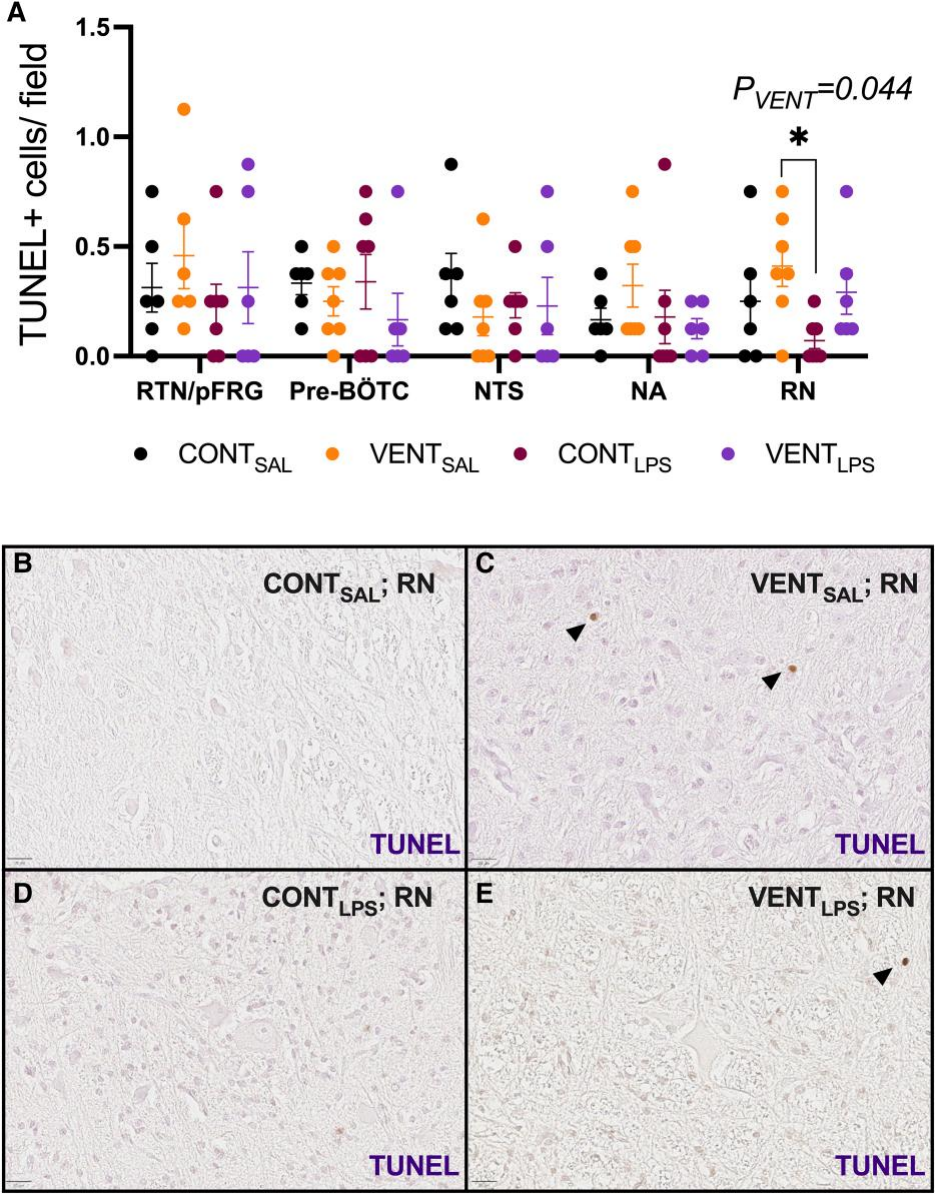
**TUNEL + cells in medullary respiration-related centres.** (**A**) terminal deoxynucleotidyl transferase-mediated dUTP nick-end labelling (TUNEL) immunopositive cells per field of view in regional respiration-related centres including the RTN/pFRG, pre-BÖTC, nucleus ambiguus (NA), nucleus tractus solitarius (NTS) and the raphe nucleus (RN), in control (CONT_SAL_; black circles; *n* = 6), ventilation + saline (VENT_SAL_; orange circles; *n* = 7), un-ventilated controls + LPS (CONT_LPS_; burgundy circles; *n* = 7) and ventilation + LPS (VENT_LPS_; purple circles; *n* = 6). Data are mean ± SEM. Two-way ANOVA with Tukey’s *pos hoc*. *P < 0.05. Each data point represents each individual foetus. (**B–E**) Representative micrographs of TUNEL+ staining in the RN in (**B**) CONT_SAL_, (**C**) VENT_SAL_, (**D**) CONT_LPS_ and (**E**) VENT_LPS_ exposed foetuses. Scale = 20 μm. Black arrowheads indicate positive staining.

The number of TUNEL+ cells/field was higher in VENT_SAL_ groups compared to CONT_LPS_ foetuses (*P* = 0.034; Fig. 7A) in the RN.

### Correlations

**Figure 8:**
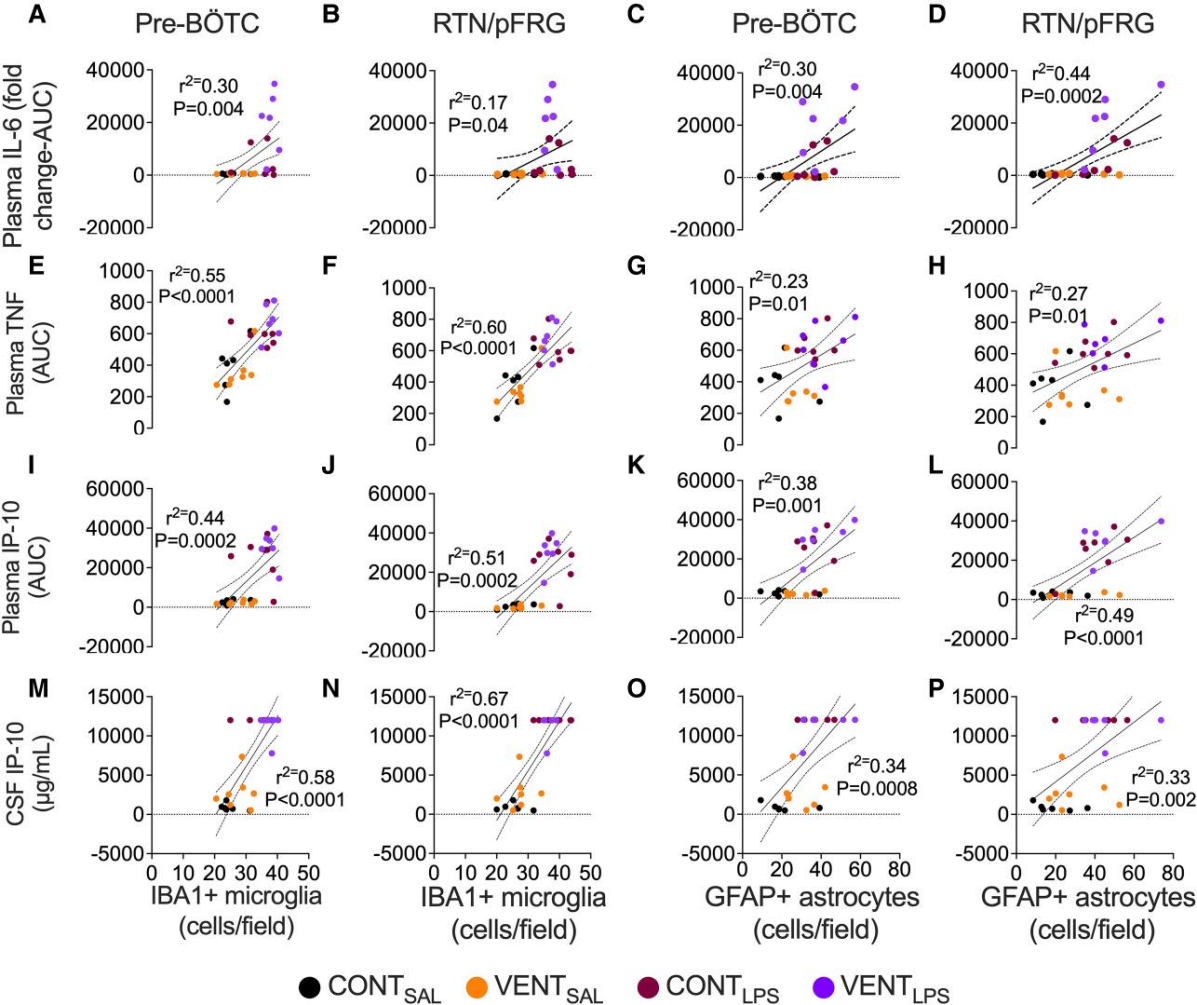
**Correlations between inflammatory proteins and IBA1+ microglia and GFAP+ astrocytes.** Correlations between total cumulative plasma protein and CSF inflammatory proteins and ionized binding adaptor (IBA1)+ microglia and glial fibrillary acidic protein (GFAP)+ astrocytes per field in control (CONT_SAL_; black circles; *n* = 6), ventilation + saline (VENT_SAL_; orange circles; *n* = 7), un-ventilated controls + LPS (CONT_LPS_; burgundy circles; *n* = 7) and ventilation + LPS (VENT_LPS_; purple circles; *n* = 6). (**A**, **E**, **I**, **M**) Correlations between IBA1+ cells of the pre-BÖTC against cumulative interleukin (IL)-6 (**A**), tumour necrosis factor (TNF) (**E**), interferon protein (IP)-10, (**I**) and CSF IP-10 (**M**). (**B**, **F**, **J**, **N**) Correlations between IBA1+ cells of the RTN/pFRG against cumulative IL-6 (**B**), TNF (**F**), IP-10 (**J**) and CSF IP-10 (**N**). (**C**, **G**, **K**, **O**) Correlations between GFAP+ cells of the pre-BÖTC against cumulative IL-6 (**C**), TNF (**G**), IP-10 (**K**) and CSF IP-10 (**O**). (**D**, **H**, **L**, **P**) Correlations between GFAP+ cells of the RTN/pFRG against cumulative IL-6 (**D**), TNF (**H**), IP-10 (**L**) and CSF IP-10 (**P**). Simple linear regression. The square of the sample correlation coefficient (*r*) is represented as *r*^2^. Each data point represents each individual foetus.

The total accumulated protein concentration of plasma IL-6 was positively correlated with the total number of IBA1+ microglia in the pre-BÖTC (*r*^2^ = 0.3, *P* = 0.004, Fig. 8A) and RTN/pFRG (*r*^2^ = 0.17, *P* = 0.014, Fig. 8B). The total accumulated plasma protein concentration of TNF was positively correlated with microglia in the pre-BÖTC (*r*^2^ = 0.55, *P* < 0.0001, Fig. 8E) and RTN/pFRG (*r*^2^ = 0.60, *P* < 0.0001, Fig. 8F). The total accumulated plasma concentration of IP-10 was positively correlated with microglia in the pre-BÖTC (*r*^2^ = 0.44, *P* = 0.0002, Fig. 8I) and the RTN/pFRG (*r*^2^ = 0.51, *P* = 0.0002, Fig. 8J). CSF concentrations of IP-10 were positively correlated (*P* < 0.0001) with IBA-1+ cells in the pre-BÖTC (*r*^2^ = 0.51, Fig. 8M) and RTN/pFRG (*r*^2^ = 0.67, Fig. 8N). The cumulative protein concentration of plasma IL-6 was positively correlated with the total number of GFAP+ astrocytes in the pre-BÖTC (*r*^2^ = 0.30, *P* = 0.004, Fig. 8C) and RTN/pFRG (*r*^2^ = 0.44, *P* = 0.0002, Fig. 8D). The cumulative protein concentration of plasma TNF was positively correlated with the total number of GFAP+ astrocytes in the pre-BÖTC (*r*^2^ = 0.23, *P* = 0.01, Fig. 8G) and RTN/pFRG (*r*^2^ = 0.27, *P* = 0.0002, Fig. 8H). Cumulative plasma IP-10 was also positively correlated with GFAP+ astrocytes of the pre-BÖTC (*r*^2^ = 0.38, *P* = 0.001, Fig. 8K) and RTN/pFRG (*r*^2^ = 0.49, *P* < 0.0001, Fig. 8L). CSF IP-10 concentration was positively correlated with the total number of GFAP+ astrocytes in the pre-BÖTC (*r*^2^ = 0.34, *P* = 0.0008, Fig. 8O) and RTN/pFRG (*r*^2^ = 0.33, *P* = 0.002, Fig. 8P).

## Discussion

Mechanical ventilation and intrauterine infection/inflammation have been shown to independently cause brainstem inflammation and impaired brainstem development in both preclinical models^16,17^ and human neonates.^15,25^ However, the effect of the combination of intrauterine infection/inflammation and low tidal volume mechanical ventilation on brainstem inflammation is poorly characterized. We investigated whether 24 h of mechanical ventilation would exacerbate brainstem inflammation and cell death following intratracheal LPS exposure. We found that LPS independently increased medullary pathological inflammation, characterized by increased numbers and activation of glial cells. The addition of ventilation following LPS administration increased plasma and CSF pro-inflammatory cytokines and brainstem *PTGS2* mRNA expression, but did not further amplify brainstem glial cell activation and inflammation compared to LPS alone. These data indicate that inflammation of the preterm foetal lungs, due to exposure to bacterial endotoxin, increases circulating inflammatory proteins and promotes an increase in markers of brainstem inflammation, which may be amplified by respiratory support.

In this study, the combination of IUV preceded by intratracheal LPS administration was associated with an increase in circulating plasma proteins IL-6, IL-10 and TNF throughout the 24-h experimental period, as well as higher IL-6 concentrations in the CSF. We have previously shown that 24 h of IUV increases mRNA expression of inflammatory genes within the lungs^35^ and causes moderate inflammation in brainstem respiratory-related centres.^16^ Despite this, 24 h of IUV did not further amplify histopathology in brainstem respiration-related centres when preceded by intratracheal LPS, suggesting that the increase in histological markers of neuroinflammation observed 24 h after mechanical ventilation was predominantly driven by LPS exposure. However, current literature suggests that the incidence of perinatal brain injury is markedly increased when insults in the immediate postnatal period, such as hypoxia-ischaemia (HI) and mechanical ventilation, are preceded by intrauterine inflammation,^43,44^ often referred to as inflammation-induced sensitization.^45,46^ A key determinant in inflammation-induced sensitization is the timing of primary and secondary insults. In a study by Eklind *et al.*,^47^ the severity of brain injury in rat pups exposed to 20 min of non-injurious HI was greater in pups exposed to LPS 4 h before HI onset compared to 2 h of LPS exposure before HI. Indeed, i.t. LPS was administered an hour before the onset of IUV, and thus, there may not have been enough time between the primary and secondary inflammatory insults to initiate an exacerbated inflammatory response in brainstem respiratory-related centres despite the exacerbated systemic inflammatory response.

The combination of mechanical ventilation and intratracheal LPS amplified plasma inflammatory proteins, strongly associated with the development of adverse neurological outcomes.^48-52^ Furthermore, CSF concentrations of IL-6 and mRNA expression of *PTGS2* were highest following the combination of ventilation and LPS. IL-6 upregulation is implicated in brain injury in infants exposed to prenatal infection/inflammation. Indeed, IL-6 concentrations were found to be elevated in the cord blood of preterm infants exposed to intrauterine inflammation and presenting postnatally with white matter lesions.^53^ IL-6 is also a critical inflammatory protein in the synthesis of prostaglandin E_2_ (PGE_2_), particularly within the CNS.^54^ PTGS2 is the rate-limiting enzyme in the production of prostaglandins. *PTGS2* mRNA upregulation is associated with astrocyte proliferation^55^ and is a potent inhibitor of breathing,^56^ inducing hypoventilation, reduced respiratory frequency and apnoeas in neonatal mice exposed to peritoneal injections of IL-1β.^57^ Elevated PGE_2_ concentrations are also associated with a reduced degree of foetal breathing movements in preterm foetal sheep infused with LPS, further suggesting that PGE_2_ is a potent inhibitor of breathing that may increase the requirement for respiratory support for longer durations.^17,24^ Despite observing increased numbers and area coverage of GFAP+ astrocytes in both LPS-exposed groups (CONT_LPS_ and VENT_LPS_), 24 h of ventilation following i.t. LPS was sufficient to increase mRNA expression of *PTGS2* in the ventilated subjects compared to controls. With the consideration that the majority of preterm infants are ventilated for longer than 24 h, we hypothesize that ventilating beyond 24 h would exacerbate histopathology in brainstem respiratory-related centres when preceded by LPS.^58^ Indeed, 24 h may have limited our ability to examine the progression of injury caused by exposure to mechanical ventilation and/or intrauterine infection/inflammation. Despite the plethora of evidence suggesting that LPS administration is the variable driving inflammation in brainstem respiratory-related centres, the number of TUNEL+ cells was higher in VENT_SAL_ compared to LPS_SAL_-exposed foetuses only in the RN. This may reflect the temporal nature of each of the insults, which will need to be addressed in future studies. Nevertheless, this study provides important insights into acute injury pathogenesis within the brainstem following a ‘double hit’ of LPS and ventilation and a platform for investigating the temporal evolution of brainstem injury in this paradigm.

Exposure to VENT_LPS_ resulted in increased PaCO_2_, decreased SaO_2_ and acidosis. We have previously shown that IUV in isolation does not affect placental function nor the foetal cardiopulmonary circulation.^16,30,35,36^ Indeed, changes in foetal biochemistry were predominantly driven by LPS administration.^50,59,60^ Inflammation is associated with increased metabolic activity, which is characterized by an increase in tissue oxygen consumption and increased CO_2_ generation.^50,60^ The addition of ventilation following i.t. LPS not only exacerbates circulating pro-inflammatory cytokines but additionally metabolic CO_2_ and oxygen demand, highlighting a synergy between both insults as previously postulated.^36^ It is possible that the greater reduction in pH, PaO_2_ and SpO_2_ following LPS exposure may contribute to the greater inflammatory response evident in brainstem respiratory-related centres in LPS-exposed foetuses, which should be addressed in future studies.

Given that the preterm lung is likely exposed to endotoxin or other pro-inflammatory products because of intrauterine infection/inflammation, we administered LPS intratracheally 1 h before the onset of ventilation to replicate a profound pro-inflammatory environment that would be at/near its peak.^61^ Direct exposure of the preterm lung to LPS amplifies lung injury caused by ventilation and causes a systemic inflammatory response.^23,62^ Moreover, it is understood that clinically, the initiation of preterm birth aligns with the peak in maternal and foetal inflammatory responses, a scenario that was most appropriately replicated by the 1 h time interval between LPS-exposure and mechanical ventilation.^21,63^ In our study, intratracheal LPS upregulated plasma pro-inflammatory cytokines compared to ventilation alone and was the primary driver of inflammation in brainstem respiration-related centres. LPS exposure has been shown to cause inflammation and injury of the white and grey matter of the cortex, which is strongly associated with the development of adverse neurological outcomes.^48-52^ However, the upregulation of plasma pro-inflammatory cytokines, including IL-1β, IL-6, IL-8 and TNF, is also associated with altered respiratory frequency and pattern generation,^17,19,64^ suggesting that the brainstem is vulnerable to the propagation of a systemic inflammatory response.^65-68^ Unique to the brainstem is the afferent arm of the inflammatory reflex. Afferent vagus nerve fibers sense peripheral inflammatory molecules, including pro- and anti-inflammatory cytokines, via mechanosensitive and chemosensitive receptors in the lung and terminate primarily within the NTS of the medulla.^69,70^ Through afferent signalling, cytokines may bypass the blood–brain barrier (whether or not affected by permeability), and signal locally within the brainstem to increase pro-inflammatory cytokine production and glial cell activation, enhancing the localized inflammatory response.^28^ It is also known that peripheral vagal afferents express toll-like receptor-4 (TLR-4), the main membrane receptor for LPS.^71^ It is therefore possible that the inflammation observed in brainstem respiration-related centres following intratracheal LPS is mediated via TLR-4-mediated vagal peripheral afferent signalling, terminating within the NTS and propagating the neuroinflammatory response from the NTS.

In the present study, LPS, both in isolation and combination with 24 h of ventilation, increased markers of astrogliosis, microgliosis and STAT3 expression within the pre-BÖTC, RTN/pFRG, NA, NTS and RN. Medullary inflammation was further evident by the higher number of morphologically amoeboid (activated) microglia within these respiratory-related centres. Microglia are the innate immune cells of the CNS and are responsible for mediating the initial immune response in the CNS following peripheral immune challenge. Activated microglia induce the further production of cytokines within the CNS to modulate both physiological and behavioural responses. The total number of microglia, as well as the number of activated microglia, was highest in LPS-exposed foetuses across all brainstem respiratory-related centres of interest. However, the number of astrocytes was increased only in the RTN/pFRG, pre-BÖTC and RN of LPS-exposed subjects. Astrocytes are activated by cytokines and play a significant role in modulating the brainstem immune response, but also in directing negative feedback to activated microglia. Astrocyte and microglial activation states can be determined by the mRNA expression of inflammatory cytokines, including *IL1B*, *TNF* and *IL10*; however, brainstem mRNA expression was taken at only one time point and was not localized to specific respiratory-related centres, therefore, STAT3 abundance was utilized as a marker of the cellular inflammatory response in the brainstem at 24 h.

Qualitatively, STAT3 expression was co-localized with microglia and astrocytes in the pre-BÖTC, indicative of glial cell activation.^72,73^ Glial cell activation is characterized by morphological and functional adaptations, which can either be neuroprotective or neurotoxic.^29,74^ Previous studies have postulated that STAT3 signalling in microglia promotes the pro-inflammatory state of activation and further release of pro-inflammatory cytokines, including IL-1β, IL-6, TNF and cyclooxygenase-2 (COX-2).^75,76^ We found an increase in the mRNA expression of *PTGS2* and *TNF* in the brainstems of foetuses exposed to ventilation and LPS. Further, CSF concentrations of pro-inflammatory cytokines IL-6 and TNF, as well as chemokine IP-10, were elevated in LPS-exposed groups (CONT_LPS_ and VENT_LPS_). Interestingly, the correlation between total microglia and plasma TNF in the pre-BÖTC (*r*^2^ = 0.55) and RTN/pFRG (*r*^2^ = 0.60) suggests that TNF is an important mediator of neuroinflammation in brainstem respiratory-related centres and may pose as a potential therapeutic target to improve brainstem inflammation and therefore cardiorespiratory function in infants born preterm that are exposed to intrauterine infection/inflammation and require prolonged periods of assisted ventilation.

Inflammation of brainstem respiratory-related centres is associated with a significant reduction in respiratory drive in animal models of intrauterine infection/inflammation.^18,57^ Specifically, the upregulation of pro-inflammatory cytokines IL-1β, IL-6 and TNF is known to alter neuronal signalling within the CNS either directly via receptor-specific expression or through glial cell activation. Previous studies have shown that astrocyte activation in the RTN/pFRG is associated with a blunted hypercapnic response and modified respiratory activity.^56,77^ It is known that IL-1β, IL-6 and TNF alter neuronal signalling through post-translational modifications of glutaminergic, GABAergic and glycinergic receptors.^29,78^ Specifically, IL-1β and TNF have been shown to decrease GABAergic and glycinergic neurotransmission and increase excitatory glutamate receptor expression.^79^ Furthermore, intravenous LPS infusion in rat pups resulted in increased extracellular glutamate and nitric oxide release in the NTS, the primary site of vagal afferent termination, only 4 h after infusion.^80^ IL-1β upregulation in the CNS results in increased excitatory neuronal output; however, IL-1β also induces the synthesis of cyclooxygenase-2 (COX-2), encoded by the *PTGS2* gene, eventuating in the production and release of prostaglandin E2 (PGE_2_).

PGE_2_ is known to play an important role in neonatal and perinatal brain injury and alter neuronal transmission in the brainstem respiration-related centres.^56^ High PGE_2_ levels are associated with hypoventilation, reduced respiratory frequency and apnoeas in neonatal mice exposed to peritoneal injections of IL1β.^57^ PGE_2_ upregulation hinders the response to hypercapnia and hypoxia, indicative of impaired central chemosensory responses in numerous animal models.^19,64^ Indeed, the increased frequency of apnoeas, hypoventilation and reduced breathing efforts in neonates exposed to intrauterine infection/inflammation and sepsis are increasingly associated with an increased requirement for respiratory support.^24,25^ In the present study, an increase in circulating inflammatory proteins, glial activation and increased *PTGS2* mRNA expression all highlight a profound inflammatory response following exposure to i.t. LPS and ventilation.

Given that preterm infants are frequently exposed to multiple inflammatory insults at birth and in the NICU while also considering the immaturity of the central control of breathing, we believe the potential for sensitization to brainstem inflammation and injury caused by exposure to multiple inflammatory triggers such as antenatal inflammation and mechanical ventilation should be an important consideration for tailoring clinical care and developing therapeutics, which target the inflammatory pathway and therefore aim to improve respiratory drive in preterm infants.

## Conclusion

This study demonstrates that 24 h of low tidal volume mechanical ventilation exacerbates systemic and brainstem neuroinflammation when preceded by intrauterine inflammation. With the consideration that preterm infants are, on average, ventilated for significantly longer periods of time than 24 h, this study provides insight that respiratory support for as little as 24 h following inflammation may adversely impact respiratory function and spontaneous breathing in preterm newborns and warrants further investigation.^58^

## Supplementary Material

fcaf441_Supplementary_Data

## Data Availability

The original contributions presented in this study are included in the article or supplementary materials. The datasets used during the current study are available from the corresponding authors upon reasonable request.

## References

[1] Garcia AJ 3rd, Zanella S, Koch H, Doi A, Ramirez JM. Chapter 3—Networks within networks: The neuronal control of breathing. Prog Brain Res. 2011;188:31–50. 21333801 10.1016/B978-0-444-53825-3.00008-5 PMC3652403

[2] Rybak IA, Abdala AP, Markin SN, Paton JF, Smith JC. Spatial organization and state-dependent mechanisms for respiratory rhythm and pattern generation. Prog Brain Res. 2007;165:201–220. 17925248 10.1016/S0079-6123(06)65013-9 PMC2408750

[3] Stojanovska V, Miller SL, Hooper SB, Polglase GR. The consequences of preterm birth and chorioamnionitis on brainstem respiratory centers: Implications for neurochemical development and altered functions by inflammation and prostaglandins. Review. Front Cell Neurosci. 2018;12:26. 29449803 10.3389/fncel.2018.00026 PMC5799271

[4] Baertsch NA, Severs LJ, Anderson TM, Ramirez J-M. A spatially dynamic network underlies the generation of inspiratory behaviors. Proc Natl Acad Sci. 2019;116(15):7493–7502. 30918122 10.1073/pnas.1900523116 PMC6462111

[5] Nuwayhid B, Brinkman CR 3rd, Su C, Bevan JA, Assali NS. Development of autonomic control of fetal circulation. Am J Physiol. 1975;228(2):337–344. 235216 10.1152/ajplegacy.1975.228.2.337

[6] Greer JJ . Control of breathing activity in the fetus and newborn. Compr Physiol. 2012;2(3):1873–1888. 23723027 10.1002/cphy.c110006

[7] Carroll JL, Agarwal A. Development of ventilatory control in infants. Paediatr Respir Rev. 2010;11(4):199–207. 21109177 10.1016/j.prrv.2010.06.002

[8] Carroll JL . Invited review: Developmental plasticity in respiratory control. J Appl Physiol. 2003;94(1):375–389. 12486025 10.1152/japplphysiol.00809.2002

[9] Beck S, Wojdyla D, Say L, et al The worldwide incidence of preterm birth: A systematic review of maternal mortality and morbidity. Bull World Health Organ. 2010;88(1):31–38. 20428351 10.2471/BLT.08.062554 PMC2802437

[10] Howson CP, Kinney MV, McDougall L, Lawn JE. Born too soon: Preterm birth matters. Reprod Health. 2013;10(Suppl 1):S1. 24625113 10.1186/1742-4755-10-S1-S1 PMC3828581

[11] Chang HH, Larson J, Blencowe H, et al Preventing preterm births: Analysis of trends and potential reductions with interventions in 39 countries with very high human development index. Lancet. 2013;381(9862):223–234. 23158883 10.1016/S0140-6736(12)61856-X PMC3572865

[12] Eichenwald EC, Committee on Fetus and Newborn, American Academy of Pediatrics. Apnea of prematurity. Pediatrics. 2016;137(1):e20153757. 10.1542/peds.2015-3757 26628729

[13] Stoll BJ, Hansen NI, Bell EF, et al Trends in care practices, morbidity, and mortality of extremely preterm neonates, 1993–2012. JAMA. 2015;314(10):1039–1051. 26348753 10.1001/jama.2015.10244 PMC4787615

[14] Gagliardi L, Bellù R, Zanini R, Dammann O. Bronchopulmonary dysplasia and brain white matter damage in the preterm infant: A complex relationship. Paediatr Perinat Epidemiol. 2009;23(6):582–590. 19840295 10.1111/j.1365-3016.2009.01069.x

[15] Guillot M, Guo T, Ufkes S, et al Mechanical ventilation duration, brainstem development, and neurodevelopment in children born preterm: A prospective cohort study. J Pediatr. 2020;226:87–95.e3. 32454115 10.1016/j.jpeds.2020.05.039

[16] Vidinopoulos K, Azman Z, Somers A, et al Mechanical ventilation induces brainstem inflammation in preterm fetal sheep. Front Pediatr. 2023;11:1225294. 37936886 10.3389/fped.2023.1225294 PMC10626530

[17] Stojanovska V, Atta J, Kelly SB, et al Increased prostaglandin E2 in brainstem respiratory centers is associated with inhibition of breathing movements in fetal sheep exposed to progressive systemic inflammation. Article. Front Physiol. 2022;13:841229. 35309054 10.3389/fphys.2022.841229 PMC8928579

[18] Siljehav V, Hofstetter AM, Leifsdottir K, Herlenius E. Prostaglandin E2 mediates cardiorespiratory disturbances during infection in neonates. J Pediatr. 2015;167(6):1207–13.e3. 26434370 10.1016/j.jpeds.2015.08.053

[19] Hofstetter AO, Saha S, Siljehav V, Jakobsson P-J, Herlenius E. The induced prostaglandin E_2_ pathway is a key regulator of the respiratory response to infection and hypoxia in neonates. Proc Natl Acad Sci. 2007;104(23):9894–9899. 17535900 10.1073/pnas.0611468104 PMC1877988

[20] Fahey JO . Clinical management of intra-amniotic infection and chorioamnionitis: A review of the literature. J Midwifery Womens Health. 2008;53(3):227–235. 18455097 10.1016/j.jmwh.2008.01.001

[21] Romero R, Espinoza J, Gonçalves LF, Kusanovic JP, Friel LA, Nien JK. Inflammation in preterm and term labour and delivery. Semin Fetal Neonatal Med. 2006;11(5):317–326. 16839830 10.1016/j.siny.2006.05.001 PMC8315239

[22] Galinsky R, Polglase GR, Hooper SB, Black MJ, Moss TJ. The consequences of chorioamnionitis: Preterm birth and effects on development. J Pregnancy. 2013;2013:412831. 23533760 10.1155/2013/412831 PMC3606792

[23] Moss TJM, Nitsos I, Kramer BW, Ikegami M, Newnham JP, Jobe AH. Intra-amniotic endotoxin induces lung maturation by direct effects on the developing respiratory tract in preterm sheep. Am J Obstet Gynecol. 2002;187(4):1059–1065. 12389005 10.1067/mob.2002.126296

[24] Panneflek TJR, Kuypers K, Polglase GR, et al The influence of chorioamnionitis on respiratory drive and spontaneous breathing of premature infants at birth: A narrative review. Eur J Pediatr. 2024;183(6):2539–2547. 38558311 10.1007/s00431-024-05508-4 PMC11098929

[25] Panneflek TJR, Kuypers KLAM, Polglase GR, Hooper SB, van den Akker T, Te Pas AB. Effect of clinical chorioamnionitis on breathing effort in premature infants at birth: A retrospective case–control study. Arch Dis Child Fetal Neonatal Ed. 2023;108(3):280–285. 36418158 10.1136/archdischild-2022-324695

[26] Frøen JF, Akre H, Stray-Pedersen B, Saugstad OD. Adverse effects of nicotine and interleukin-1beta on autoresuscitation after apnea in piglets: Implications for sudden infant death syndrome. Pediatrics. 2000;105(4):e52. 10742373 10.1542/peds.105.4.e52

[27] Hofstetter AO, Herlenius E. Interleukin-1beta depresses hypoxic gasping and autoresuscitation in neonatal DBA/1lacJ mice. Respir Physiol Neurobiol. 2005;146(2-3):135–146. 15766902 10.1016/j.resp.2004.11.002

[28] Balan KV, Kc P, Mayer CA, Wilson CG, Belkadi A, Martin RJ. Intrapulmonary lipopolysaccharide exposure upregulates cytokine expression in the neonatal brainstem. Acta Paediatr. 2012;101(5):466–471. 22176020 10.1111/j.1651-2227.2011.02564.x PMC4132655

[29] Vezzani A, Viviani B. Neuromodulatory properties of inflammatory cytokines and their impact on neuronal excitability. Neuropharmacology. 2015;96(Pt A):70–82. 25445483 10.1016/j.neuropharm.2014.10.027

[30] Allison BJ, Crossley KJ, Flecknoe SJ, et al Ventilation of the very immature lung in utero induces injury and BPD-like changes in lung structure in fetal sheep. Pediatr Res. 2008;64(4):387–392. 18552709 10.1203/PDR.0b013e318181e05e

[31] Chan KY, Tran NT, Papagianis PC, et al Investigating pathways of ventilation induced brain injury on cerebral white matter inflammation and injury after 24 h in preterm lambs. Front Physiol. 2022;13:904144. 35860659 10.3389/fphys.2022.904144 PMC9289398

[32] Allison BJ, Crossley KJ, Flecknoe SJ, Morley CJ, Polglase GR, Hooper SB. Pulmonary hemodynamic responses to in utero ventilation in very immature fetal sheep. Respir Res. 2010;11(1):111. 20723253 10.1186/1465-9921-11-111 PMC2944277

[33] Barton SK, Tolcos M, Miller SL, et al Unraveling the links between the initiation of ventilation and brain injury in preterm infants. Front Pediatr. 2015;3:97. 26618148 10.3389/fped.2015.00097 PMC4639621

[34] Percie du Sert N, Hurst V, Ahluwalia A, et al The ARRIVE guidelines 2.0: Updated guidelines for reporting animal research. PLoS Biol. 2020;18(7):e3000410. 32663219 10.1371/journal.pbio.3000410 PMC7360023

[35] Azman Z, Vidinopoulos K, Somers A, et al In utero ventilation induces lung parenchymal and vascular alterations in extremely preterm fetal sheep. Am J Physiol Lung Cell Mol Physiol. 2024;326:L330–L343. 38252635 10.1152/ajplung.00249.2023

[36] Tran NT, Somers A, Vidinopoulos K, et al The synergistic effects of mechanical ventilation and intrauterine inflammation on cerebral inflammation in preterm fetal sheep. Front Cell Neurosci. 2024;18:1397658. 38962513 10.3389/fncel.2024.1397658 PMC11220153

[37] Keszler M, Nassabeh-Montazami S, Abubakar K. Evolution of tidal volume requirement during the first 3 weeks of life in infants <800 g ventilated with volume guarantee. Arch Dis Child Fetal Neonatal Ed. 2009;94(4):F279–F282. 19060010 10.1136/adc.2008.147157

[38] Stojanovska V, Atik A, Nitsos I, et al Effects of intrauterine inflammation on cortical gray matter of near-term lambs. Front Pediatr. 2018;6:145. 29963540 10.3389/fped.2018.00145 PMC6013568

[39] Bankhead P, Loughrey MB, Fernández JA, et al Qupath: Open source software for digital pathology image analysis. Sci Rep. 2017;7(1):16878. 29203879 10.1038/s41598-017-17204-5 PMC5715110

[40] Pekny M, Pekna M. Astrocyte reactivity and reactive astrogliosis: Costs and benefits. Physiol Rev. 2014;94(4):1077–1098. 25287860 10.1152/physrev.00041.2013

[41] Kettenmann H, Hanisch UK, Noda M, Verkhratsky A. Physiology of microglia. Physiol Rev. 2011;91(2):461–553. 21527731 10.1152/physrev.00011.2010

[42] Nott F, Jane Pillow J, Dahl M, et al Brain inflammation and injury at 48 h is not altered by human amnion epithelial cells in ventilated preterm lambs. Pediatr Res. 2020;88:27–37. 32120374 10.1038/s41390-020-0815-8

[43] Hagberg H, Gressens P, Mallard C. Inflammation during fetal and neonatal life: Implications for neurologic and neuropsychiatric disease in children and adults. Ann Neurol. 2012;71(4):444–457. 22334391 10.1002/ana.22620

[44] Galinsky R, Lear CA, Dean JM, et al Complex interactions between hypoxia-ischemia and inflammation in preterm brain injury. Dev Med Child Neurol. 2018;60(2):126–133. 29194585 10.1111/dmcn.13629

[45] Eklind S, Mallard C, Leverin A-L, et al Bacterial endotoxin sensitizes the immature brain to hypoxic–ischaemic injury. Eur J Neurosci. 2001;13(6):1101–1106. 11285007 10.1046/j.0953-816x.2001.01474.x

[46] Stridh L, Mottahedin A, Johansson ME, et al Toll-like receptor-3 activation increases the vulnerability of the neonatal brain to hypoxia-ischemia. J Neurosci. 2013;33(29):12041–12051. 23864690 10.1523/JNEUROSCI.0673-13.2013 PMC3713735

[47] Eklind S, Mallard C, Arvidsson P, Hagberg H. Lipopolysaccharide induces both a primary and a secondary phase of sensitization in the developing rat brain. Pediatr Res. 2005;58(1):112–116. 15879289 10.1203/01.PDR.0000163513.03619.8D

[48] Bose CL, Laughon MM, Allred EN, et al Systemic inflammation associated with mechanical ventilation among extremely preterm infants. Cytokine. 2013;61(1):315–322. 23148992 10.1016/j.cyto.2012.10.014 PMC3518391

[49] Galinsky R, Dhillon SK, Dean JM, et al Tumor necrosis factor inhibition attenuates white matter gliosis after systemic inflammation in preterm fetal sheep. J Neuroinflammation. 2020;17(1):92. 32293473 10.1186/s12974-020-01769-6 PMC7087378

[50] Galinsky R, van de Looij Y, Mitchell N, et al Magnetic resonance imaging correlates of white matter gliosis and injury in preterm fetal sheep exposed to progressive systemic inflammation. Int J Mol Sci. 2020;21(23):8891. 33255257 10.3390/ijms21238891 PMC7727662

[51] Leviton A, Kuban K, O'Shea TM, et al The relationship between early concentrations of 25 blood proteins and cerebral white matter injury in preterm newborns: The ELGAN study. J Pediatr. 2011;158(6):897–903.e1–5. 21238986 10.1016/j.jpeds.2010.11.059

[52] Kelly SB, Stojanovska V, Zahra VA, et al Interleukin-1 blockade attenuates white matter inflammation and oligodendrocyte loss after progressive systemic lipopolysaccharide exposure in near-term fetal sheep. J Neuroinflammation. 2021;18(1):189. 34465372 10.1186/s12974-021-02238-4 PMC8408978

[53] Yoon BH, Romero R, Yang SH, et al Interleukin-6 concentrations in umbilical cord plasma are elevated in neonates with white matter lesions associated with periventricular leukomalacia. Am J Obstet Gynecol. 1996;174(5):1433–1440. 9065108 10.1016/s0002-9378(96)70585-9

[54] Nilsberth C, Elander L, Hamzic N, et al The role of interleukin-6 in lipopolysaccharide-induced fever by mechanisms independent of prostaglandin E2. Endocrinology. 2009;150(4):1850–1860. 19022895 10.1210/en.2008-0806

[55] Zhou J, Geng Y, Su T, et al NMDA receptor-dependent prostaglandin-endoperoxide synthase 2 induction in neurons promotes glial proliferation during brain development and injury. Cell Rep. 2022;38(13):110557. 35354047 10.1016/j.celrep.2022.110557

[56] Forsberg D, Ringstedt T, Herlenius E. Astrocytes release prostaglandin E2 to modify respiratory network activity. eLife. 2017;6:e29566. 28976306 10.7554/eLife.29566 PMC5648524

[57] Siljehav V, Shvarev Y, Herlenius E. Il-1β and prostaglandin E_2_ attenuate the hypercapnic as well as the hypoxic respiratory response via prostaglandin E receptor type 3 in neonatal mice. J Appl Physiol. 2014;117(9):1027–1036. 25213632 10.1152/japplphysiol.00542.2014

[58] Malhotra A, Castillo-Melendez M, Allison BJ, et al Neuropathology as a consequence of neonatal ventilation in premature growth-restricted lambs. Am J Physiol Regul Integr Comp Physiol. 2018;315(6):R1183–R1194. 30230932 10.1152/ajpregu.00171.2018

[59] Dalitz P, Harding R, Rees SM, Cock ML. Prolonged reductions in placental blood flow and cerebral oxygen delivery in preterm fetal sheep exposed to endotoxin: Possible factors in whitge matter injury after acute infection. J Soc Gynecol Investig. 2003;10(5):283–290. 10.1016/s1071-5576(03)00090-x 12853089

[60] Galinsky R, Hooper SB, Polglase GR, Moss TJ. Intrauterine inflammation alters fetal cardiopulmonary and cerebral haemodynamics in sheep. J Physiol. 2013;591(20):5061–5070. 23878364 10.1113/jphysiol.2013.259119 PMC3810809

[61] Kramer BW, Ikegami M, Jobe AH. Intratracheal endotoxin causes systemic inflammation in ventilated preterm lambs. Am J Respir Crit Care Med. 2002;165(4):463–469. 11850337 10.1164/ajrccm.165.4.2011118

[62] Polglase GR, Hillman NH, Ball MK, et al Lung and systemic inflammation in preterm lambs on continuous positive airway pressure or conventional ventilation. Pediatr Res. 2009;65(1):67–71. 18704000 10.1203/PDR.0b013e318189487e

[63] Goldenberg RL, Hauth JC, Andrews WW. Intrauterine infection and preterm delivery. N Engl J Med. 2000;342(20):1500–1507. 10816189 10.1056/NEJM200005183422007

[64] Guerra FA, Savich RD, Wallen LD, et al Prostaglandin E2 causes hypoventilation and apnea in newborn lambs. J Appl Physiol. 1988;64(5):2160–2166. 3164715 10.1152/jappl.1988.64.5.2160

[65] Zeni P, Doepker E, Schulze-Topphoff U, Huewel S, Tenenbaum T, Galla HJ. MMPs contribute to TNF-alpha-induced alteration of the blood-cerebrospinal fluid barrier in vitro. Am J Physiol Cell Physiol. 2007;293(3):C855–C864. 17507431 10.1152/ajpcell.00470.2006

[66] Tilling T, Korte D, Hoheisel D, Galla HJ. Basement membrane proteins influence brain capillary endothelial barrier function in vitro. J Neurochem. 1998;71(3):1151–1157. 9721740 10.1046/j.1471-4159.1998.71031151.x

[67] Malaeb S, Dammann O. Fetal inflammatory response and brain injury in the preterm newborn. J Child Neurol. 2009;24(9):1119–1126. 19605775 10.1177/0883073809338066 PMC3695470

[68] Vitkovic L, Konsman JP, Bockaert J, Dantzer R, Homburger V, Jacque C. Cytokine signals propagate through the brain. Mol Psychiatry. 2000;5(6):604–615. 11126391 10.1038/sj.mp.4000813

[69] Goehler LE, Gaykema RP, Hansen MK, Anderson K, Maier SF, Watkins LR. Vagal immune-to-brain communication: A visceral chemosensory pathway. Auton Neurosci. 2000;85(1-3):49–59. 11189026 10.1016/S1566-0702(00)00219-8

[70] Lee LY, Pisarri TE. Afferent properties and reflex functions of bronchopulmonary C-fibers. Respir Physiol. 2001;125(1-2):47–65. 11240152 10.1016/s0034-5687(00)00204-8

[71] Jia L, Lee S, Tierney JA, Elmquist JK, Burton MD, Gautron L. TLR4 signaling selectively and directly promotes CGRP release from vagal afferents in the mouse. eNeuro. 2021;8(1):ENEURO.0254-20.2020. 10.1523/ENEURO.0254-20.2020 PMC7877464 33318075

[72] Zheng ZV, Chen J, Lyu H, et al Novel role of STAT3 in microglia-dependent neuroinflammation after experimental subarachnoid haemorrhage. Stroke Vasc Neurol. 2022;7(1):62–70. 34645687 10.1136/svn-2021-001028 PMC8899684

[73] Kim JA, Yun H-M, Jin P, et al Inhibitory effect of a 2,4-bis(4-hydroxyphenyl)-2-butenal diacetate on neuro-inflammatory reactions via inhibition of STAT1 and STAT3 activation in cultured astrocytes and microglial BV-2 cells. Neuropharmacology. 2014;79:476–487. 23891616 10.1016/j.neuropharm.2013.06.032

[74] Gao C, Jiang J, Tan Y, Chen S. Microglia in neurodegenerative diseases: Mechanism and potential therapeutic targets. Signal Transduct Target Ther. 2023;8(1):359. 37735487 10.1038/s41392-023-01588-0 PMC10514343

[75] Park J, Lee C, Kim Y. Effects of natural product-derived compounds on inflammatory pain via regulation of microglial activation. Pharmaceuticals. 2023:16:941. 37513853 10.3390/ph16070941 PMC10386117

[76] Haim LB, Ceyzériat K, Carrillo-de Sauvage MA, et al The JAK/STAT3 pathway is a common inducer of astrocyte reactivity in Alzheimer's and Huntington's diseases. J Neurosci. 2015;35(6):2817–2829. 25673868 10.1523/JNEUROSCI.3516-14.2015 PMC6605603

[77] Forsberg D, Horn Z, Tserga E, et al CO_2_-evoked release of PGE2 modulates sighs and inspiration as demonstrated in brainstem organotypic culture. eLife. 2016:5:e14170. 27377173 10.7554/eLife.14170 PMC4974055

[78] Galic MA, Riazi K, Pittman QJ. Cytokines and brain excitability. Front Neuroendocrinol. 2012;33(1):116–125. 22214786 10.1016/j.yfrne.2011.12.002 PMC3547977

[79] Zipp F, Bittner S, Schafer DP. Cytokines as emerging regulators of central nervous system synapses. Immunity. 2023;56(5):914–925. 37163992 10.1016/j.immuni.2023.04.011 PMC10233069

[80] Lin HC, Wan FJ, Kang BH, Wu CC, Tseng CJ. Systemic administration of lipopolysaccharide induces release of nitric oxide and glutamate and c-fos expression in the nucleus tractus solitarii of rats. Hypertension. 1999;33(5):1218–1224. 10334815 10.1161/01.hyp.33.5.1218

