# Supplementary material for: Mechanical ventilation amplifies intratracheal lipopolysaccharide-induced plasma and brainstem inflammation in preterm foetal sheep
Source: Brain Commun. 2025 Nov 14;7(6):fcaf441. doi: 10.1093/braincomms/fcaf441 (PMC12670003; doi:10.1093/braincomms/fcaf441)
Supplement: fcaf441_Supplementary_Data [file fcaf441_supplementary_data.pdf]

Supplementary figures

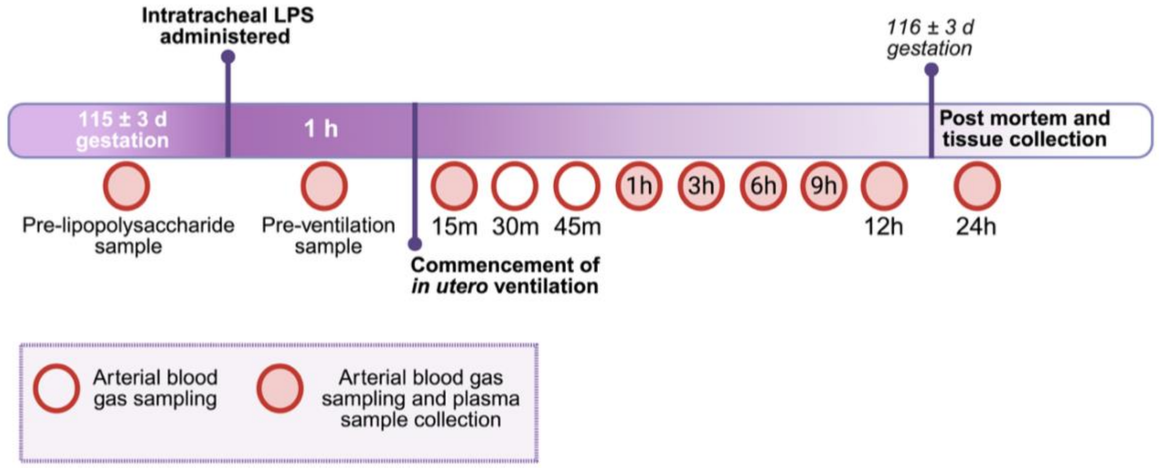

Supplementary figure 1. Experimental design and timeline. Experimental protocols began at 115 ± 3 days of gestation (d) and were completed at 116 ± 3d. During the 24 h ventilation protocol, arterial blood gas measurements and plasma sample collection occurred at baseline (pre- LPS), 1 h following LPS (pre-ventilation), 15 minutes (m), 1, 3, 6, 9, 12, and 24 h (red circles) after ventilation initiation. Additional arterial blood gas samples were taken at 30 and 45 m after *in utero* ventilation (IUV) initiation. Following the 24 h of IUV or equivalent control period, ewes and fetuses were euthanised and tissue was collected for analysis. Created in BioRender. *Vidinopoulos, K. (2025). https://BioRender.com/ms9chzc*

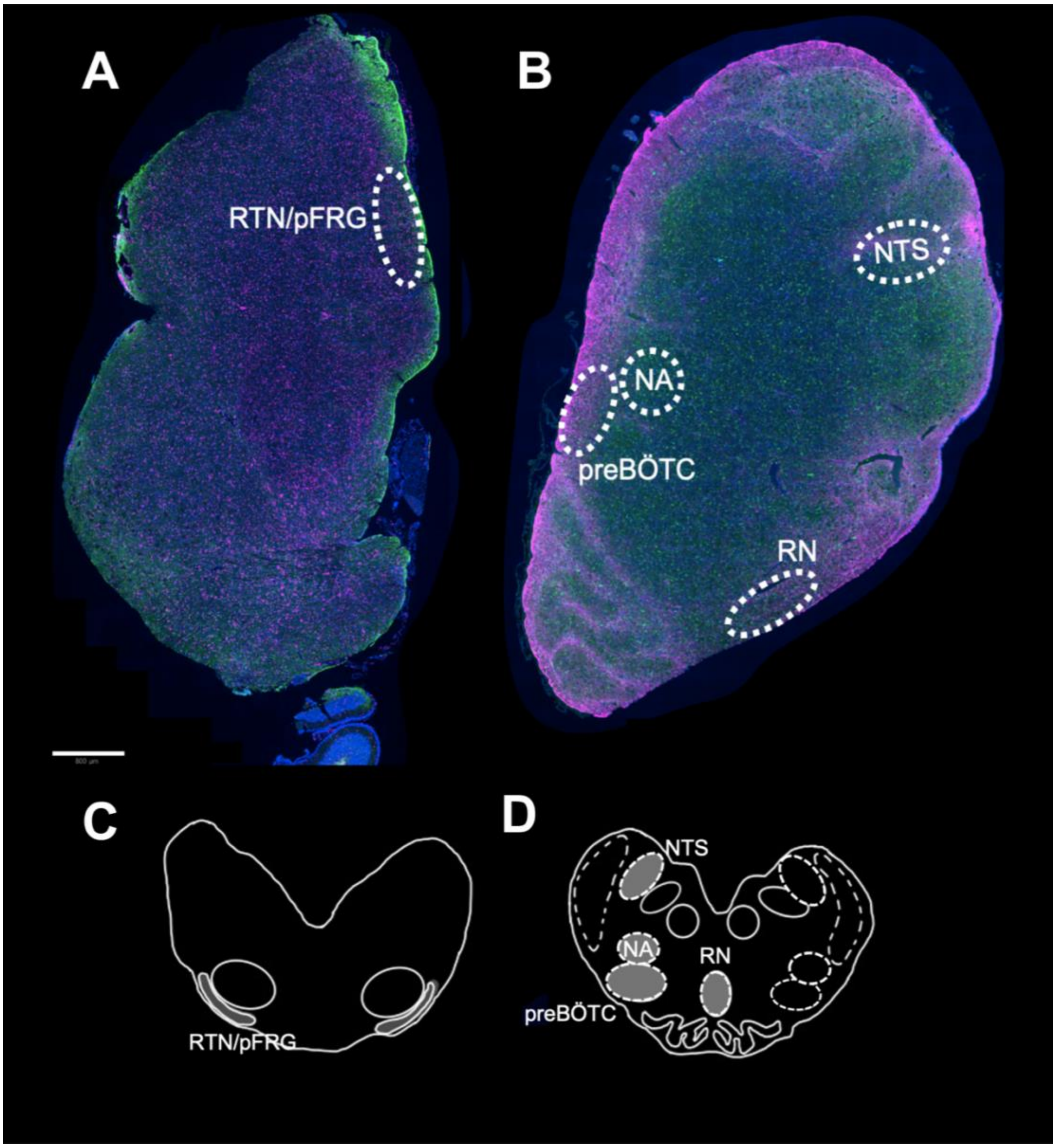

Supplementary figure 2. Schematic representation of the approximate location in the medulla of the respiratory centres of interest that were analysed including the retrotrapezoid nucleus and parafacial respiratory group (RTN/pFRG; panels A and C) and the preBötzinger complex (pre-BÖTC), the nucleus tractus solitarius (NTS), the raphe nucleus (RN) and the nucleus ambiguus (NA; panels B and D). Blue indicates positive nuclei staining (HOECHST), magenta indicates positive astrocyte staining (glial fibrillary acidic protein (GFAP), 647), and green indicates positive microglial staining (ionized calcium binding molecule (IBA-1), 488). Scale bar= 800µm.

Supplementary table 1. List of TaqMan gene array ovine-specific probes for qPCR

Biological Process	Gene name	Genes/ Probe	Assay ID
Inflammation	Interleukin 1 alpha	*IL-1A*	Oa04658682_m1
	Interleukin 1 beta	*IL-1B*	Oa04656322_m1
	Interleukin 6	*IL-6*	Oa04656315_m1
	Interleukin 8	*IL-8*	Bt03211906_m1
	Interleukin 10	*IL-10*	Oa03212724_m1
	Interleukin 18	*IL-18*	Oa04658606_m1
	Tumour necrosis factor	*TNF*	Oa04656867_g1
	Nuclear factor kappa B	*NFκB*	Oa04837805_m1
	CXC motif chemokine ligand 10	*CXCL-10*	Oa04655788_g1
	High mobility group box 1	*HMGB1*	Ch04812286_s1
	Forkhead box P3	*FOXP3*	Oa03233950_g1
	Metallopeptidase 9	*MMP-9*	Oa03215996_g1
	Myeloperoxidase	*MPO*	Oa04654413_g1
	Serum amyloid A	*SAA*	Oa04924154_s1
	Toll-like receptor 4	*TLR-4*	Oa04656419_m1
	Prostaglandin E synthase	*PTGES*	Oa04920211_s1
	Prostaglandin endoperoxide synthase 2	*PTGS-2*	Oa04657348_g1
Cell injury/ death	Caspase 1	*CASP-1*	Oa04775045_m1
	Caspase 3	*CASP-3*	Oa04817361_m1
	Caspase 8	*CASP-8*	Oa04779925_m1
	Heat shock protein 70	*HSP70*	Oa04849683_g1
Housekeeping genes	Ribosomal protein S18	*18S*	Oa4906333_g1
	Beta 2 microglobulin	*B2M*	Oa04900279_Mh
	Ribosomal protein S29	*S29*	Ch04807765_gH

Supplementary table 2. Fetal characteristics and baseline arterial blood gas parameters. Fetal sheep characteristics at post-mortem and fetal arterial blood gas parameters at baseline between control (CONTSAL; n=6), ventilation + saline (VENTSAL; n=7), unventilated controls + LPS (CONTLPS; n=7), and ventilation + LPS (VENTLPS; n=6). Data are mean ± SEM. Groups that do not share a common letter are significantly different ($P<0.05$) from each other.

	**$CONT_{SAL}$**	**$VENT_{SAL}$**	**$CONT_{LPS}$**	**$VENT_{LPS}$**
Number (n)	6	7	7	6
Body weight (kg)	2.7 ± 0.4	2.7 ± 0.5	2.7 ± 0.1	2.8 ± 0.2
Sex (% male)	67	57	27	17
Twin (%)	83	57	100	83
Brain weight (g)	36.3 ± 2	35.8 ± 1	39.3 ± 2	36.6 ± 3

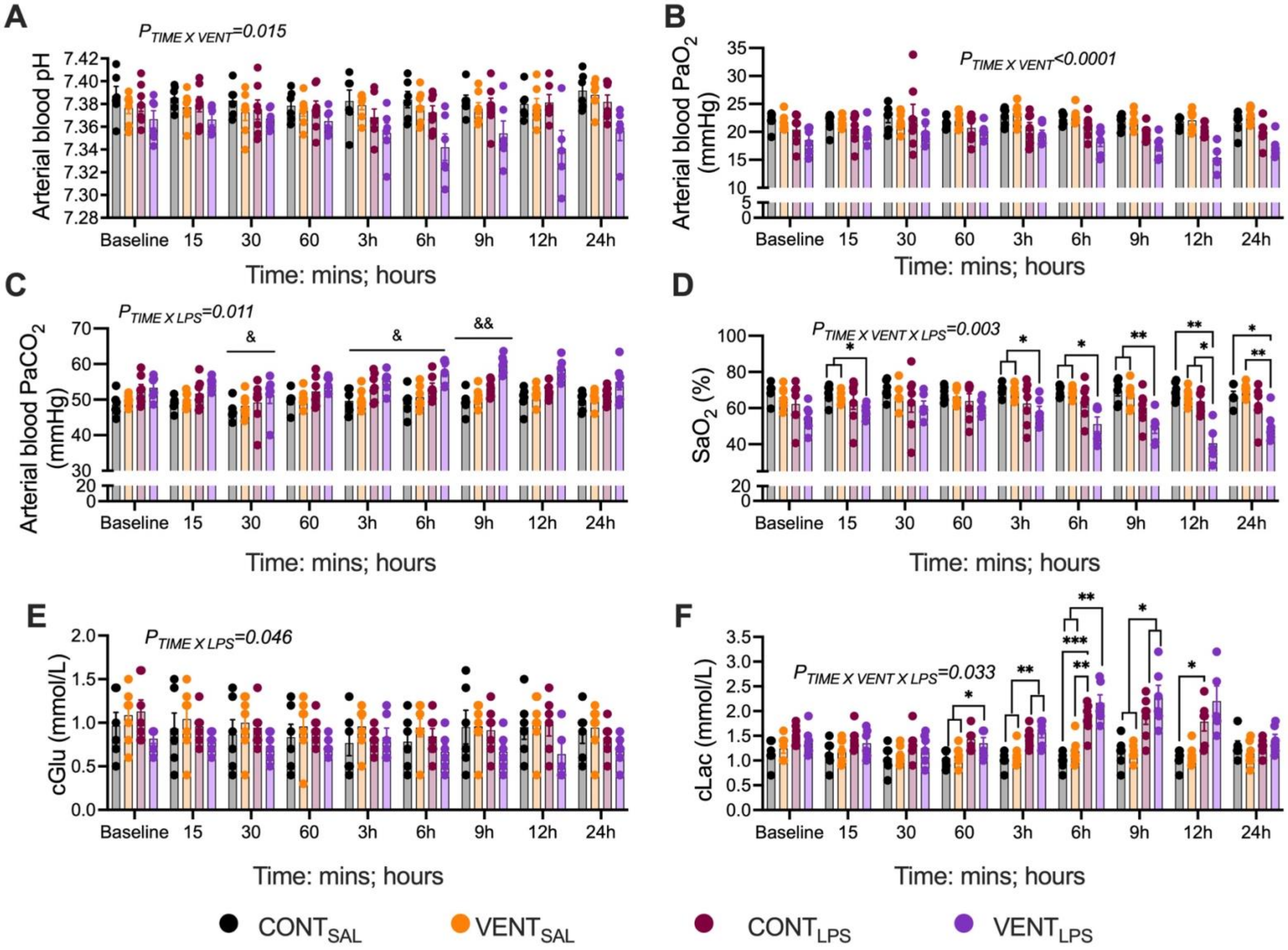

Supplementary figure 3. Fetal biochemistry. Arterial blood (A) pH, (B) partial pressure of O_2 (PaO_2 (mmHg)), (C) partial pressure of CO_2 ($PaCO_2$ (mmHg)), (D) oxygen saturation (SaO_2(%)), (E) glucose (cGlu (mmol/L)), and (F) lactate (cLac (mmol/L)) taken from baseline (pre-LPS) and throughout 24 hours of ventilation from control (CONT$_{SAL}$; black circles; n=6), ventilation + saline (VENT$_{SAL}$; orange circles; n=7), un-ventilated controls + LPS (CONT$_{LPS}$; burgundy circles; n=7) and ventilation + LPS (VENT$_{LPS}$; purple circles; n=6). Data are mean ± SEM. Mixed effect with Tukey's post-hoc differences between groups indicated as *p<0.05, **p<0.01 and ***p<0.001; Two-way ANOVA with Sidak's post-hoc to determine differences between CONT v LPS indicated as $^{\&}$p<0.05, $^{\&\&}$p<0.01. Each datapoint represents individual fetuses.
